# Critical role of alpha spectrin in DNA repair: the importance of μ-calpain and Fanconi anemia proteins

**DOI:** 10.3389/ebm.2025.10537

**Published:** 2025-05-01

**Authors:** Muriel W. Lambert

**Affiliations:** Department of Pathology, Immunology and Laboratory Medicine, Rutgers New Jersey Medical School, Newark, NJ, United States

**Keywords:** alpha spectrin, DNA repair, DNA interstrand crosslinks, μ-calpain, Fanconi anemia proteins

## Abstract

Nonerythroid spectrins are proteins important in maintaining the structural integrity and flexibility of the cell and nuclear membranes and are essential for a number of functionally important cellular processes. One of these proteins, nonerythroid α spectrin (αSpII), plays a critical role in DNA repair, specifically repair of DNA interstrand crosslinks (ICLs), where it acts as a scaffold, recruiting repair proteins to sites of damage. Loss or breakdown of αSpII is an important factor in a number of disorders. One of these is Fanconi anemia (FA), a genetic disorder characterized by bone marrow failure, chromosome instability, cancer predisposition, congenital abnormalities and a defect in DNA ICL repair. Significantly, breakdown of αSpII occurs in cells from a number of FA complementation groups, due to excessive cleavage by the protease, μ-calpain, leading to defective repair of DNA ICLs in telomeric and non-telomeric DNA. Knockdown of μ-calpain in FA cells by μ-calpain siRNA results in restoration of αSpII levels to normal and repair of DNA ICLs in telomeric and non-telomeric DNA, demonstrating the importance of αSpII stability in the repair process. It is hypothesized that there is a mechanistic link between excessive cleavage of αSpII by μ-calpain and defective DNA ICL repair in FA and that FA proteins, which are deficient in FA, play a key role in maintaining the stability of αSpII and preventing its cleavage by μ-calpain. All of these events are proposed to be important key factors involved in the pathophysiology of FA and suggest new avenues for potential therapeutic intervention.

## Impact statement

In the nucleus, the structural protein nonerythroid α spectrin (αSpII) plays a central role in repair of DNA interstrand crosslinks (ICLs). Significantly, there is a deficiency in αSpII in the genetic, bone marrow failure disorder, Fanconi anemia (FA), which is characterized by a defect in DNA ICL repair. This is due to excessive cleavage of αSpII by the protease, µ-calpain. Importantly, knockdown of µ-calpain, by siRNA, reverses cleavage of αSpII, restores its levels to normal and enables repair of DNA ICLs in FA cells. FA thus represents another disorder in which excessive cleavage of αSpII by µ-calpain correlates with a major characteristic, in this case defective DNA ICL repair. It demonstrates the importance of αSpII for the DNA ICL repair process. FA proteins are proposed to play a major role in regulating cleavage of αSpII by µ-calpain thus giving them another critical role in DNA repair.

## Introduction

Cytoskeletal and nucleoskeletal proteins are critical for maintaining the structure, function and mechanical properties of eukaryotic cells [1–11]. Of particular importance is spectrin, found in both erythroid and nonerythroid cells [12–19]. Spectrin was first identified in erythrocytes and shown to be essential for cell membrane structure, integrity, and flexibility [14, 15, 18, 20–24]. Spectrin also plays a critical role in non-erythroid cells in both the cytoplasm and nucleus and is involved in maintaining the shape, flexibility and elasticity of both the cell and nuclear membranes and is essential for a number of functionally important cytoplasmic and nuclear processes, as well as for mechanical coupling between the nucleus and the cytoplasm [7, 10, 13–17, 25–39]. Total loss of spectrin can lead to cell death [33, 36, 38, 40]. Nonerythroid spectrin has two subunits, αSpII and βSpII [14–17, 34, 35]. Of considerable interest, nuclear αSpII has been shown to directly interact with DNA and play a critical role in DNA repair and in the maintenance of the stability of chromosomes and telomeres after DNA damage [12, 13, 38, 41–46]. It acts as a scaffold and aids in recruitment of repair proteins to sites of damage in both telomeric and non-telomeric DNA [42–49].

Loss or breakdown of spectrin in cells leads to a deficiency in these processes and is clinically manifested in a number of disorders, one of which is the genetic disorder, Fanconi anemia (FA) [13, 38, 44, 48, 50]. FA is characterized by bone marrow failure, a marked propensity to develop cancer, multisystemic congenital abnormalities, chromosomal instability and a defect in ability to repair DNA interstrand cross-links (ICLs) [51–69]. Lambert et. al. have shown a deficiency in αSpII in cells from FA patients from a number of FA complementation groups [12, 13, 44, 48]. This deficiency is due to the excessive activity of the protease, µ-calpain, which cleaves αSpII leading to its breakdown [69, 70]. The breakdown of αSpII correlates with a defect in DNA ICL repair in FA cells in both nontelomeric and telomeric DNA and to chromosome instability [46, 69]. These studies have demonstrated that maintaining the stability of αSpII in the cell is critical for a number of important nuclear and cellular processes and for circumventing telomere dysfunction after DNA ICL damage [46, 69]. Lambert et al. have proposed that excessive cleavage of αSpII by μ-calpain is an important factor in the pathogenesis of FA and a number of the clinical characteristics of this disorder [13, 50, 69].

This review will concentrate on the function of αSpII in the nucleus with emphasis on its interaction with non-telomeric and telomeric DNA, especially after DNA ICL damage, the importance of maintaining the stability of αSpII and preventing its breakdown by μ-calpain, and the role of FA proteins in this process. The consequences of a loss or deficiency in αSpII on DNA repair, telomere integrity/function and chromosome stability after DNA damage, particularly in FA, will be discussed.

## Overview of spectrin structure

**FIGURE 1:**
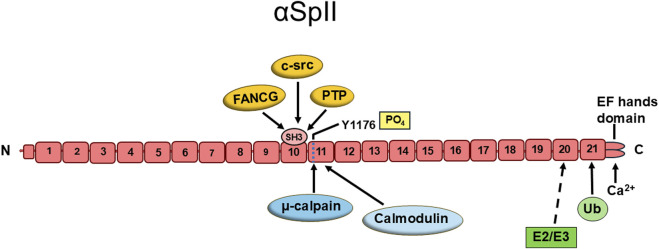
The structure and domains of human non-erythroid alpha spectrin (αSpII). αSpII is composed of 21 triple helical repeats. Repeat 10 contains a Src-homology 3 (SH3) domain which is a site of protein-protein interactions. Three proteins targeting this site are: c-src, a kinase; low molecular weight phosphotyrosine phosphatase (PTP); and FANCG. Repeat 11 has a site of cleavage by the protease, μ-calpain, and also contains a binding domain for calmodulin. There is a suggested E2/E3 ubiquitin protein conjugating/ligating site (E2/E3) in repeat 20 and a ubiquitination site (Ub) in repeat 21. At the C-terminus of αSpII are two ER-hand domains which bind calcium. Domains 20 and 21 mediate the dimerization of αSpII at its C-terminus with the N-terminus of βSpII. This structure of αSpII is based on studies and models of a number of investigators. [14–17, 25, 74–77].

αSpII is a long flexible protein which contains a modular structure composed of an extended array of triple α-helical repeats connected by short a-helical linker [14–17, 21, 27, 71–73]. This structure aids in its flexibility and ability to expand and contract [14, 25, 71–73]. Nonerythroid spectrin has two subunits, αSpII and βSpII. There is one alpha spectrin gene and four beta spectrin genes [14–17]. There are a number of different spectrin isoforms which originate by extensive mRNA splicing from these genes [14, 27]. In all of these, αSpII and βSpII associate in an antiparallel fashion to form a heterodimer [14–17]. αSpII consists of 21 triple helical repeats and βSpII contains 17 repeats [14–17]. Two heterodimers can link head to head to form a tetramer [14–17]. Nonerythroid spectrin (αSpII/βSpII) is comprised of numerous domains. αSpII contains an EF hand’s domain on repeat 21, which is a site of Ca^2+^ binding and signaling (Figure 1) [14–17]. It contains a Src-homology 3 (SH3) domain in repeat 10, which plays an important role in proteinprotein interactions and is involved in signal transduction and intracellular signaling (Figure 1) [14–17, 74, 78–81]. The SH3 domain has a binding site for a kinase, c-Src, lowmolecular weight phosphotyrosine phosphatase (LMW-PTP) and FANCG, a Fanconi anemia (FA) protein. αSpII has a site of cleavage by the protease, calpain, in repeat 11 between residues Y_1176_ and G_1177._ [75, 76] There is a caspase cleavage site at residue Asp1185, which is activated during apoptosis [82]. αSpII also has a calmodulin binding domain in repeat 11, which can modulate spectrin cleavage by µ-calpain and caspase [83, 84]. Proteolytic cleavage of αSpII by µ-calpain leads to its decreased stability and loss of function, which makes regulation of µ-calpain cleavage of αSpII critical for maintaining normal cell and nuclear functioning [74, 76, 78]. There is also a ubiquitination site in repeat 21 and a suggested E2/E3-ubiquitin-conjugating/ligating site in repeat 20 [14, 26]. βSpII has an actin-binding domain on repeat 1, and a pleckstrin homology domain on repeat 7, which is involved in cell signaling, organization of the cytoskeleton, and aiding in the localization of βSpII to the plasma membrane [14–17]. αSpII and βSpII assemble laterally to form heterodimers that assemble into tetramers [14–17]. The tertiary structure of the spectrin repeat imparts elasticity to the protein [14–17, 21, 27, 71–73]. The structural characteristics of αSpII/βSpII enable it to interact with a number of different cellular proteins and participate in a number of important physiological pathways in the cell in both the nucleus and the cytoplasm. Numerous reviews are available with a moredetailed description of spectrin structure [14–17, 20, 24–27, 71–73].

## Spectrin and its role in the cytoplasm

Spectrin is a multifunctional protein, as is evidenced by the numerous proteins which interact with both αSpII and βSpII. Goodman and colleagues have created the Spectrinome using Human Proteome, Human Reactome and Human Atlas data which clearly demonstrates the multitude of cellular pathways and functions in which spectrin is involved in the cytoplasm, the nucleus and the cell surface [14]. Since spectrin’s numerous interacting protein partners demonstrate the multitude of roles spectrin plays in the cell, it is important to more fully understand these interactions and their impact on cellular function.

**FIGURE 2:**
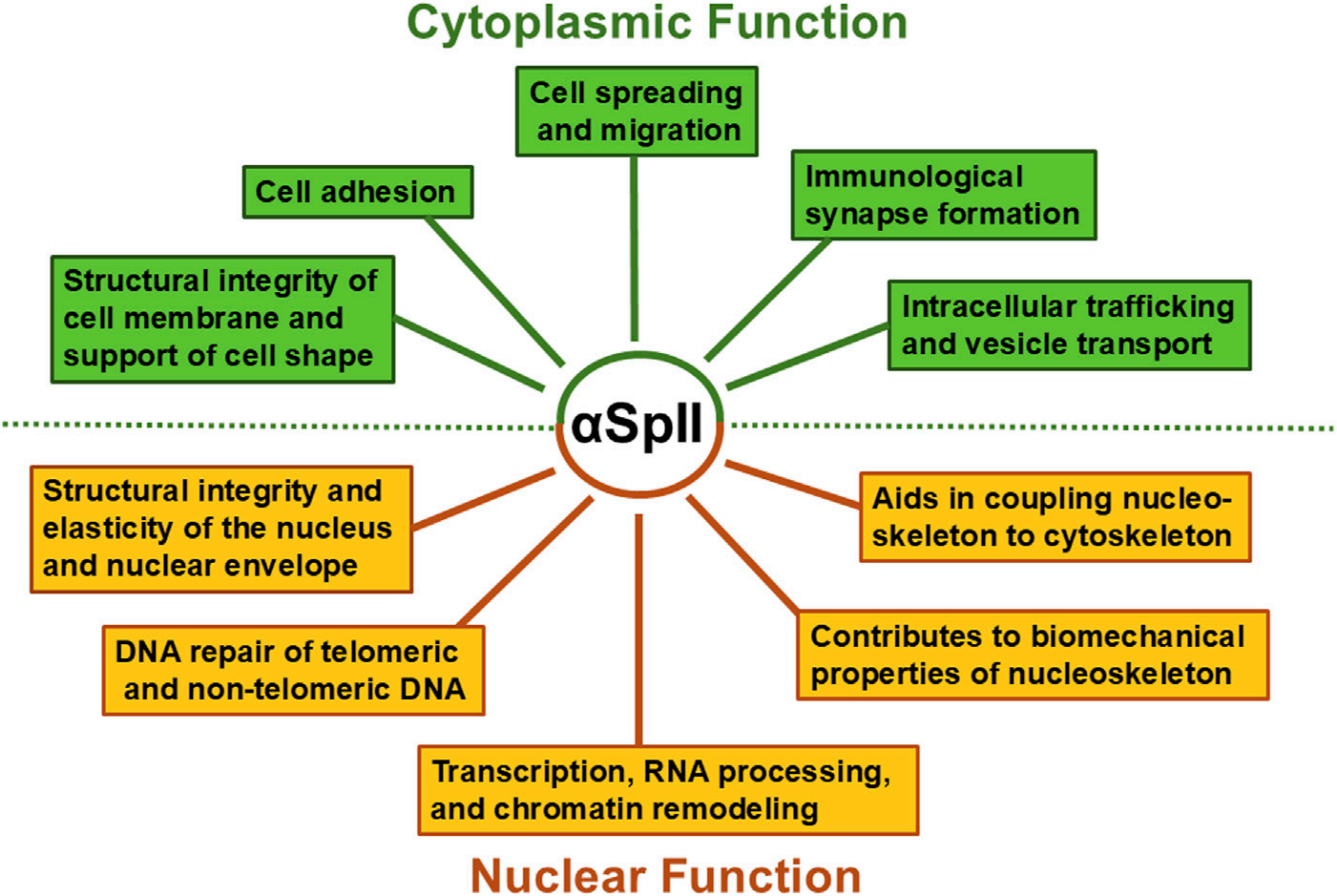
Roles for αSpII in the nucleus and cytoplasm. αSpII is involved in multiple functions in both the nucleus and cytoplasm of human non-erythroid cells and thus has an important impact on a number of different cellular pathways.

One of the best known and critical roles of αSpII/βSpII is providing structural integrity for cell membranes [14, 19, 85, 86]. It acts as a scaffold interacting with ankyrin, actin and other cytoskeletal proteins to form a network crucial for maintaining mechanical support, shape, flexibility and elasticity to the cell membrane [17, 24, 85–89]. αSpII is involved in cell movement and adhesion via interactions with proteins involved in actin dynamics (Figure 2) [31–33, 89]. αSpII plays a role in actin filament reorganization which is critical for a number of processes such as lamellipodia extension and immunological synapse formation between T-cells and antigen-presenting cells [27, 32, 33, 89]. The SH3 domain of αSpII has been shown to bind to proteins involved in actin polymerization and reorganization and plays a crucial role in this process during cell-cell contact, cell adhesion, cell spreading and migration [29, 33, 89–91]. Spectrin interacts with proteins involved in intracellular traffic. It is involved in vesicle and organelle mediated transport [14, 92]. In neurons, αSpII/βSpII and αSpII/βSpIII have an essential role in synaptic vesicle trafficking and synaptic transmission [14, 16, 93]. βSpIII associates with the microtubule motor proteins, kinesin and dynein, and is involved in anterograde and retrograde transport of cargo in axons [14, 94–96]. αSpII and βSpII associate with synaptic vesicles via synapsin I and are critical for this process [14, 28, 29, 97]. αSpII and βSpII have been identified associated with melanosomes in human melanocytes and are thought to be involved in melanosome transport in these cells [98]. Goodman et al have also shown that erythroid spectrin has E2/E3 chimeric ubiquitin conjugating and ligating activity and is capable of ubiquitinating not only alpha spectrin but also ankyrin, band 3, and protein 4.1 [14, 26, 99, 100]. Goodman et al. have proposed that αSpII has similar E2/E3 ubiquitinating activity in the cytoplasm and the nucleus in non-erythroid cells [14, 26, 99, 100]. This activity is very important for protein-protein interactions and the role they play in normal physiological processes in the cell. Thus, αSpII along with βSpII are components of a spectrin scaffold which is critical for a large number of physiological processes in the cytoplasm.

## Spectrin and its structural role in the nucleus

Just as interactions of spectrin with specific proteins in the cytoplasm are critical for cellular function, its interactions with both proteins and DNA in the nucleus is critical for nuclear function. Within the nucleus, nonerythroid spectrin is a component of a nucleoskeletal network which plays an essential role in maintaining the structural integrity and elasticity of the nucleus as well as in repair of damage to both nontelomeric and telomeric DNA (Figure 2) [7, 10, 15–17, 27, 37, 46, 50, 101]. Both αSpII and two isoforms of β-spectrin (βSpII and βSpIV∑5) have been identified in the nucleus [12, 13, 38, 39]. Studies have shown that αSpII is present in the peripheral nucleoskeleton, where it interacts with lamins A and B, actin, and nuclear myosin and is anchored to the nuclear envelope by emerin and protein 4.1 [7, 8, 10, 13, 37, 41, 50, 102]. It makes an important contribution to the biomechanical properties of these nucleoskeleton proteins and to nuclear envelope support [7, 10, 37, 102]. It forms a complex with lamins A and B, actin, nuclear myosin, and the LINC complex protein, SUN2, and plays an important role in coupling the nucleoskeleton to the cytoskeleton [7, 10, 13, 37, 41]. αSpII is also associated with the inner nucleoskeleton where it interacts with DNA repair proteins, chromatin remodeling proteins and proteins involved in transcription and RNA processing [6–8, 13, 41, 42].

The cortical network αSpII forms with lamin and actin at the nuclear envelop plays a role in maintaining the elasticity and structural integrity of the nucleus (Figure 2) [7, 10, 15, 17, 27, 37]. Knock-down of αSpII in HeLa cells to levels that are approximately 50% of those found in control cells leads to a decrease in ability of the nucleus to recover from compression [37]. This has been attributed to loss of membrane elasticity due to decreased αSpII [37]. αSpII’s modular structure of triple alpha-helical repeats makes an important contribution to the structural stability, elasticity and mechanical resilience of the nucleus and to its ability to recover from compression [37].

Thus, αSpII is an important scaffolding protein which represents 2–3% of the total protein in eukaryotic cells [14]. It has a myriad of functions and any defects or deficiencies in this protein would be central to pathological changes occurring in the cell structure and function (Figure 2). Therefore, maintaining αSpII stability is critical for normal cell functioning. Excessive cleavage of αSpII can upset normal cellular homeostasis and result in various human disease states and disorders. These points will be discussed below.

## αII spectrin plays a critical role in DNA repair in the nucleus

In addition to its structural role in the nucleus, αSpII has been shown to have an essential function in the nucleus in repair of DNA damage. Lambert and colleagues have demonstrated that in human cells αSpII plays a critical role in repair of DNA interstrand cross-links (ICLs) in both non-telomeric and telomeric DNA and is needed for chromosomal stability after DNA damage [13, 42–49, 69]. There are numerous lines of evidence which demonstrate the important role of αSpII in DNA repair: (1) αSpII binds directly to DNA at sites of ICLs; (2) αSpII recruits repair proteins to these sites; (3) αSpII is needed for production of incisions produced by the endonuclease XPF-ERCC1 at sites of ICLs; and (4) αSpII is critical for repair of telomeric DNA and telomere integrity after DNA ICL damage [42–46, 69]. Thus, αSpII is important in DNA repair processes in the nucleus and maintenance of telomere integrity after DNA damage. These points will be discussed below.

### αSpII localizes to sites of DNA ICLs and binds directly to DNA at these sites

Lambert *et al.* have demonstrated that αSpII from HeLa cell nuclei, as well as purified bovine brain αSpII, bind directly to a DNA substrate containing a 4,5′8-trimethylpsoralen (TMP) plus UVA light induced ICL [42]. Based on the crystal structure of αSpIl, it has been proposed that it binds to the minor groove of DNA which opens up after ICL formation [42]. αSpII’s binding to the damaged DNA is specific for the ICL; it does not bind to DNA containing a TMP monoadduct [42]. In HeLa and human lymphoblastoid cells damaged with a DNA ICL agent, 8-methoxysporalen (8-MOP) plus UVA light or mitomycin C (MMC), αSpII localizes in damage induced nuclear foci which are sites of DNA ICLs [42–46, 49, 69]. These studies provide strong evidence that αSpII plays a role in the damage recognition steps of the DNA ICL repair process.

### αSpII is needed for recruitment of XPF-ERCC1 and repair proteins to sites of ICLs and production of incisions at these sites

**FIGURE 3:**
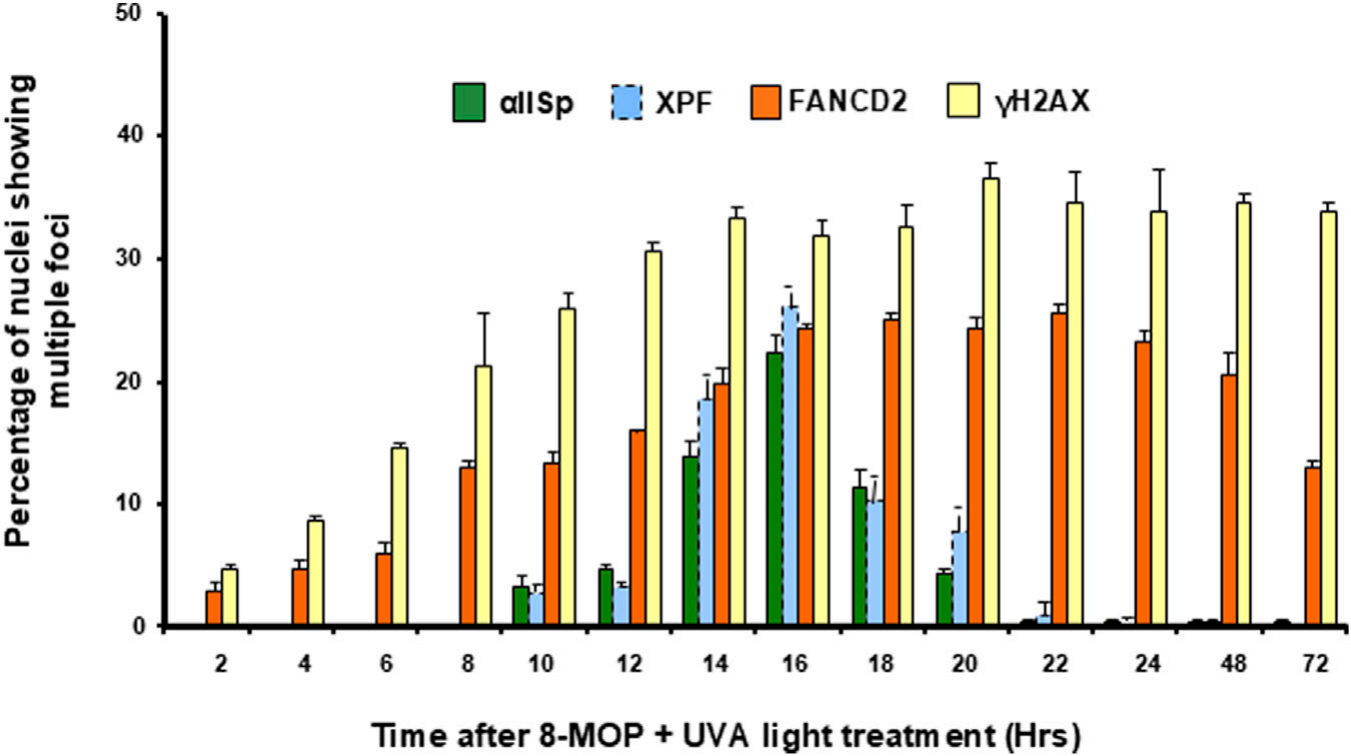
Time course for appearance of αSpII, XPF, FANCD2, and γ -H2AX nuclear foci after damage of normal human cells with a DNA ICL agent. The percentage of nuclei showing multiple αSpII, XPF, FANCD2, and γ -H2AX foci was determined at time points from 0 to 72 h after treatment with 8-MOP plus UVA light. Nuclei containing four or more foci were counted as positive. αSpII foci co-localized with XPF foci and had a similar time course after damage of cells with 8-MOP plus UVA light. These foci appeared after FANCD2 foci and had a different time course than the FANCD2 foci. Error bars represent SEM. (Reproduced from Zhang et al., [49] with permission from John Wiley and Sons).

αSpII colocalizes in damaged-induced nuclear foci with proteins involved in DNA ICL repair. These include FANCA, FANCF, FANCG, and XPF-ERCC1 [42–46, 69]. XPF-ERCC1 is involved in the unhooking of the ICL and production of endonucleolytic incisions at the site of the ICL [48, 103–106]. Lambert and colleagues demonstrated that, after normal human lymphoblastoid cells or HeLa cells are damaged with a DNA ICL agent, αSpII colocalizes with XPF at damage-induced nuclear foci [43, 46, 69]. Time course studies have shown that αSpII co-localizes with XPF at the same nuclear foci, with foci first appearing 10 h after ICL damage with 8-MOP plus UVA light or MMC (Figure 3) [13, 43]. Foci peak at 16h and disappear by 24h when ICLs are no longer present [13, 43]. The same time course and co-localization observed with αSpII and XPF was also observed with the repair proteins FANCA and FANCF [13, 43]. This indicates that both αSpII and XPF are involved in the same events in DNA ICL repair and suggests that FANCA and FANCF may also play a role.

Additional evidence that αSpII is critical for recruitment of XPF and these repair proteins to sites of ICLs has been demonstrated by immunofluorescence studies in which αSpII has been knocked down by siRNA [45, 46]. Since αSpII is an essential protein for cell survival, total loss of this protein results in cell death [14, 107, 108]. Cells survive, however, when levels are reduced to 35–40% of normal [45, 46]. Reduction of αSpII to these levels followed by damaging DNA with an ICL agent has been shown to result in decreased formation of damage-specific αSpII foci in these cells and loss of localization of XPF to the sites of damage [45]. FA proteins are present in these cells and FANCA and FANCF also localize to the sites of damage with XPF [45]. Thus, αSpII is needed for the recruitment and localization of XPF as well as FANCA and FANCF to sites of DNA ICLs indicating their involvement in ICL repair. Corroborating these findings are studies which have shown that inhibition of expression of the αSpII gene, *SPTAN1*, by miR-125-3p, leads to disruption of localization of XPF and FANCA to mitomycin C induced foci after treatment of lung cancer cells with this DNA interstrand cross-linking agent [109]. This has been suggested as a novel pathway for interrupting repair of DNA ICLs and enhancing the anticancer function of DNA ICL agents [109].

Further support for the importance of αSpII for incisions produced by XPF-ERCC1 is provided by studies which have demonstrated that, on a DNA substrate containing a site-specific tri-methylpsoralen (TMP) ICL, XPF-ERCC1 produces incisions at the site of a DNA ICL [42]. Purified bovine αSpII enhances these incisions but does not itself produce incisions at the site of the ICL [42]. A purified monoclonal antibody against αSpII from normal human lymphoblastoid cells inhibited these incisions [42, 48]. Thus the presence of αSpII is critical for the incisions produced by XPF-ERCC1 at sites of ICLs. The studies described above have led Lambert and colleagues to develop a model in which αSpII acts as a scaffold to aid in recruitment of repair proteins such as XPF-ERCC1 to sites of DNA ICLs; loss of αSpII results in a deficiency in recruitment of XPF and decreased incisions at sites of ICLs leading to a deficiency in DNA repair [42].

### The SH3 domain of αSpII is an important site of recruitment for XPF-ERCC1 and repair proteins to sites of DNA ICLs

An important question is by what mechanism does αSpII recruit repair proteins to sites of ICLs? As mentioned above, αSpII consists of 21 triple-helical repeats [14–17]. There is a Src-homology 3 (SH3) domain in the 10th repeat (Figure 1) [14–17, 78]. SH3 domains are modular domains which are important in protein-protein interactions and protein network assembly [79, 110]. There are three major classes of protein ligands which bind to SH3 domains, class I, class II and class I@ [74, 79–81]. The SH3 domain of αSpII has a consensus sequence which preferentially binds to class 1@ ligands [74]. A number of FA proteins have consensus sequences that bind to SH3 domains and have the potential to bind to cellular proteins containing these domains [101]. Of particular interest, FANCG has a class1@ consensus sequence [101]. Lambert et al have carried out studies using sitedirected mutagenesis and yeast two hybrid analysis and have shown that FANCG binds directly to the SH3 domain of αSpII via this consensus sequence [101]. FANCG also binds directly to XPF-ERCC1 [101]. FANCG contains seven tetratricopeptide repeat (TPR) motifs, which are motifs that are involved in protein-protein interactions [111–114]. TPR motifs 1, 2, 3, and 6 in FANCG bind directly to the central domain of ERCC1 (residues 120–220) [115]. ERCC1 binds to XPF by its C-terminal domain (residues 220–297) [116–118]. XPF in turn binds to ERCC1 through its C-terminal domain, which differs from its nuclease domain, and produces incisions at the sites of DNA ICLs [118, 119]. Thus the SH3 domain of αSpII plays an important role in the ability of αSpII to recruit XPF-ERCC1, via FANCG, to sites of ICLs, enabling it to create incisions at the site of damage. Knocking down αSpII eliminates its binding to sites of DNA ICLs and recruitment of XPF-ERCC1 to these sites [45, 46]. This, in turn, results in reduction in production of incisions on damaged DNA [45, 46]. Whether αSpII is involved in additional steps in the repair process has not yet been determined.

### A model for the role of αSpII in the DNA ICL repair pathway

Repair of DNA ICLs can occur in both replicating and non-replicating DNA [106, 120–125]. This repair process is particularly important in S phase of the cell cycle when DNA replication is stalled at the site of an ICL. Stalled DNA replication forks can trigger DNA ICL repair, thus making replication-coupled ICL repair extremely critical for cell survival [106, 120–125]. Several models have been proposed for repair of ICLs at stalled replication forks. Three of the major pathways for replication-coupled ICL repair are: (1) The Fanconi anemia pathway, which is triggered when two replication forks converge on an ICL. This pathway involves a large number of proteins and a complex series of steps resulting in production of incisions in DNA and unhooking of the ICL; (2) The Neil3 pathway, in which ICLs are resolved by a DNA glycosylase and cleavage of one of the two N-glycosyl bonds forming the ICL; and (3) The acetaldehyde pathway, which denotes a mechanism for repair of acetaldehyde-induced ICLs [106, 125]. In some instances unhooking involves incision of the ICL via the FA pathway and in others the ICL may undergo enzymatic reversal [106, 125].

Replication-coupled ICL repair has been extensively studied in cells from patients with Fanconi anemia (FA) who are deficient in ability to repair DNA interstrand crosslinks [57–61, 106, 123, 124]. FA is a genetic disorder, which, in addition to defective DNA ICL repair, is characterized by bone marrow failure, congenital abnormalities, chromosome instability and a predisposition to develop a variety of cancers [51, 52, 57, 61–68]. There are 22 different FA genes (*FANCA* to *FANCW*). Germline mutations in any one of these genes can cause the disorder. The FANC proteins expressed by these genes are all involved in replication-coupled DNA repair and the pathway in which they are involved is known as the Fanconi anemia (FA) pathway [57–61, 104, 126–131]. Since αSpII has been shown to play a critical role in ICL repair during S phase of the cell cycle, to directly interact with proteins in the FA pathway, and to be important in the initial damage recognition and incision steps of the ICL repair process, the involvement of αSpII in this pathway will be described [13, 42–46].

**FIGURE 4:**
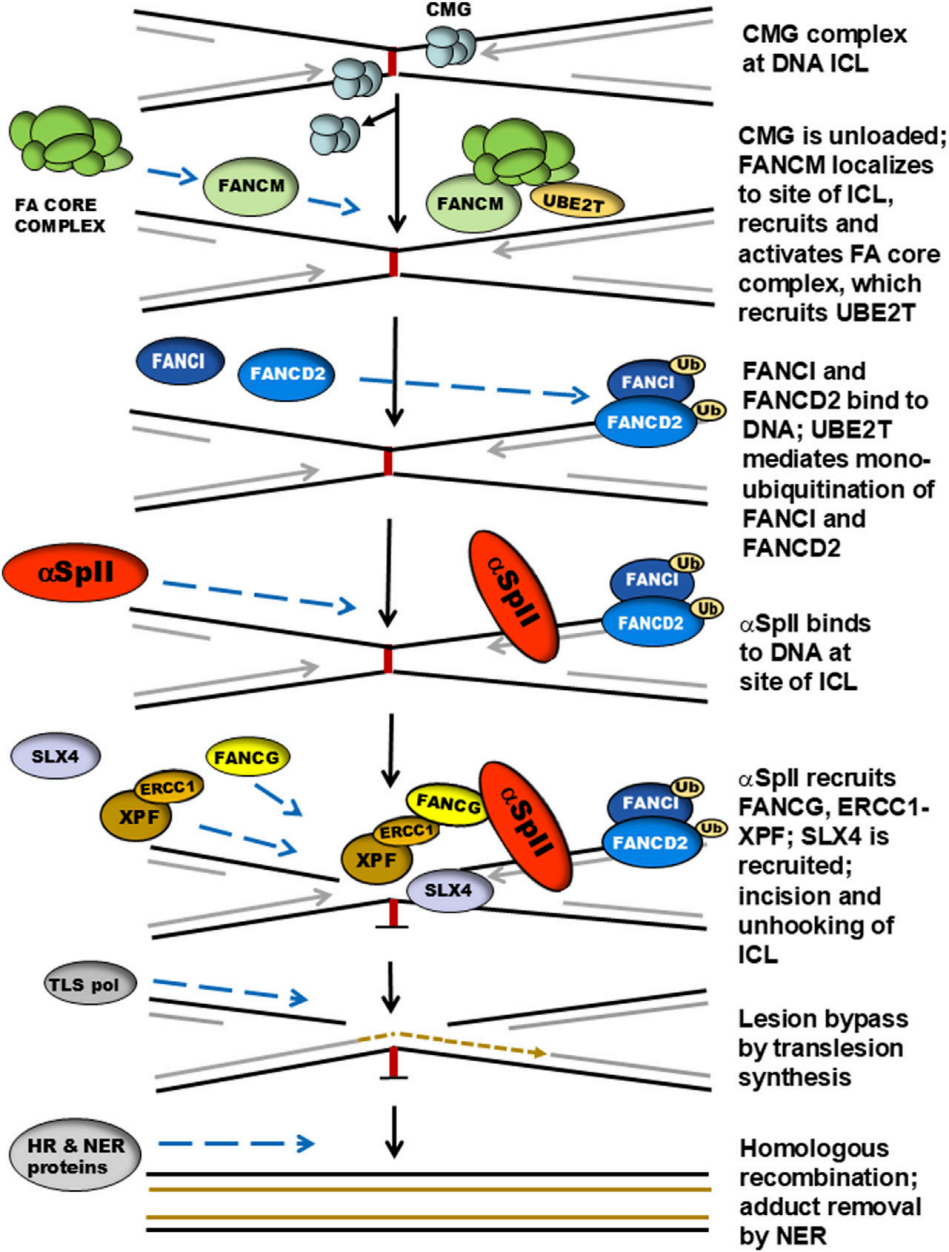
A model for the role of αIISp in replication-coupled DNA ICL repair. When DNA replication is stalled at the site of an ICL and replication forks converge, the CMG helicase complex on the leading strand stalls and is unloaded. FANCM recognizes the stalled replication fork and localizes to the damaged DNA. The FANCM complex recruits the FA core complex. FANCD2 and FANCI localize to the site of damage at the stalled replication fork. The core complex recruits UBE2T (FANCT), which monoubiquitinates FANCD2 and FANCI. αSpII subsequently binds to DNA at the site of the ICL downstream of FANCD2-Ub and acts as a scaffold aiding in the recruitment of repair proteins to the site of damage. FANCG binds to the SH3 domain of αSpII and XPF-ERCC1 is recruited and binds to FANCG-αSpII. XPF then produces incisions at the site of damage and is involved in the unhooking of the ICL. SLX4/FANCP plays a role in this step. Translesion DNA synthesis then occurs by a translesion polymerase leading to bypassing the ICL. This is followed by additional steps which include homologous recombination (HR) and adduct removal by nucleotide excision repair (NER). This model emphasizes the role of αSpII in the initial damage-recognition and incision steps of the DNA ICL repair process. It is based on studies on the interaction of αSpII with DNA containing ICLs, on the involvement of numerous proteins involved in the repair process and on other models for ICL repair [42–44, 50, 57, 66, 104, 109, 127, 132–135]. (Modified from Lambert [50], with permission of Sage Publications Ltd.).

The initial damage recognition step of the ICL repair process is critical. In replication-coupled DNA ICL repair, the site of damage is located at a stalled replication fork [57–59, 66, 106, 121]. Lambert and colleagues have proposed a model for the mechanism of action of αSpII in ICL repair in the FA repair pathway (Figure 4) [13, 50]. It is based on studies on the interaction of αSpII with DNA containing ICLs and with proteins involved in the ICL repair process and on previous models for ICL repair [13, 50, 57, 66, 106, 121, 124, 136]. Although αSpII is not a FANC protein, it plays a critical role in this pathway [13, 50]. In this pathway, replication forks converge at the site of an ICL and the CDC45/MCM2-7/GINS (CMG) helicase complex on the leading strand is unloaded [66, 106, 126, 130]. FANCM and a group of interacting proteins recognize the stalled replication fork and localize to the DNA [57, 66, 106, 125, 127, 131]. The FANCM complex helps recruit the FA core complex (FANCA, FANCB, FANCC, FANCE, FANCF, FANCG, FANCL, FAAP20 and FAAP100), which has E3 ubiquitin ligase activity [57, 66, 106, 125, 131]. FANCD2/FANCI localize at the replication fork stalled at the site of the ICL [106, 136–139]. The core complex recruits a ubiquitinconjugating enzyme, UBE2T (FANCT), which monoubiquitinates FANCI and FANCD2 (the ID complex) (Figure 4). [106, 125, 136–139] αSpII subsequently binds to the site of an ICL on DNA downstream of FANCD2 [18, 50].

After αSpII binds to the DNA, it aids in the recruitment of XPF-ERCC1 to the site of the ICL via recruitment of FANCG [18, 50]. As described above, FANCG directly binds to the SH3 domain of αSpII via its class 1@ consequence sequence and recruits XPFERCC1 [101]. The central domain of ERCC1 binds to TPR motifs 1, 2, 3, and 6 in FANCG; the C-terminal domain of XPF binds to ERCC1 [115–118]. XPF then creates an incision at the site of the ICL leading to unhooking of the ICL [57, 66, 103]. Another endonuclease, SLX4/FANCP is also involved in the incision process and aids in protein coordination [57, 66, 104, 128, 129]. Thus, αSpII recognizes and binds to DNA at sites of ICLs and acts as a scaffold in recruitment of XPF-ERCC1 via FANCG. It then incises the DNA and is involved in the unhooking of the ICL. SLX4/FANCP is recruited and also participates in this step [104–106]. Other FA proteins could be involved in this repair process as well.

After these events, DNA translesion synthesis takes place and the lesion is bypassed by a translesion DNA polymerase [58, 66, 106, 124, 125]. This is followed by homologous recombination and adduct removal by NER [58, 66, 106, 124, 125]. A large number of FA proteins take part in these steps. These stages in the repair process, which involve DNA translesion synthesis, homologous recombination and NER are discussed in detail in numerous reviews and will not be elaborated upon here [57–61, 66, 106, 124–126]. The reader is directed to these reviews. Since the role of αSpII in ICL repair is mainly in the initial damage recognition and incision steps of the repair pathway, these are the components of the FA pathway that have been emphasized here.

The exact relationship between αSpII and FANCD2 in this repair process is not clear. Time course experiments show that αSpII binds to the damaged DNA after FANCD2 (Figure 4) [49]. It does not colocalize with monoubiquitinated FANCD2 (FANCD2Ub) in nuclear foci nor is it required for monoubiquitination of FANCD2 after ICL damage or for its localization to sites of damage [49]. Monoubiquitination of FANCD2 is not required for binding of αSpII to damaged DNA [49]. The time course for localization of FANCD2 to nuclear foci after ICL damage is different than that of αSpII (Figure 3) [49]. FANCD2 foci first appear 2 h after ICL damage, increase up until 16 h, remain fairly stable until 24 h and are still present at 72 h (Figure 3) [49]. In contrast, αSpII foci first appear 10 h after DNA ICL formation, peak at 16 h and disappear by 24 h [49]. Thus, FANCD2-Ub associates in foci with damaged DNA before αSpII foci appear and is still present when αSpII foci are gone [49]. The time course of FANCD2 foci corresponds to that of formation of ɣH2AX (phosphorylated histone H2A) foci which localize to sites of DNA double strand breaks (Figure 3) [49]. Both FANCD2 and ɣH2AX foci are present 72 h after damage, which could indicate additional involvement of FANCD2 in later stages in the ICL repair process as has been proposed [49, 50, 136]. The relationship between αSpII and ubiquitinated FANCD2 needs to be further explored.

In human cells, αSpII is also needed for the localization of FANCA and FANCF to sites of ICL damage [43]. FANCA and FANCF do not localize to nuclear foci after ICL damage if αSpII has been knocked down by siRNA [45]. Whether this colocalization of FANCA and FANCF with αSpII and XPF at damage sites is a component of the recruitment of the FA core complex to sites of DNA damage or is a component of the steps involved in the unhooking of the DNA ICL is not clear and needs to be further investigated.

## Importance of μ-calpain and FA proteins in the regulation of cleavage of αSpII in FA cells

The importance of αSpII stability in DNA repair is seen in cells from patients with Fanconi anemia (FA) which, as mentioned above, are defective in ability to repair DNA ICLs [43–48, 51–61]. Of significance, Lambert et. al. have shown that there is a deficiency in αSpII in cells from patients from a number of FA complementation groups [12, 13, 45, 69]. Levels of αSpII are 35–40% of those found in normal human cells [12, 13, 45]. This deficiency correlates with reduced levels of repair of DNA ICLs in FA cells which are 35–45% of normal [45, 47, 48]. Levels of DNA repair after DNA ICL damage were determined in FA-A, FA-C, FA-D2, FA-F and FA-G cells by measurement of unscheduled DNA synthesis (UDS), which measures uptake of nucleotides into DNA repair patches [47].

An important question is, what is the cause of the reduced levels of αSpII in FA cells? Lambert et al have shown that this is not due to reduced expression of αSpII, since FA cells express normal levels of αSpII mRNA [70]. They have demonstrated that it is due to increased cleavage of αSpII as a result of excessive activity of the protease, μcalpain, which cleaves αSpII [69]. They have proposed that FA proteins play a critical role in regulation of μ-calpain cleavage of αSpII and that reduced levels of FA proteins in FA cells leads to increased μ-calpain activity and increased cleavage of αSpII [50, 69]. Thus, both increased μ-calpain activity and reduced levels of FA proteins are proposed to play a critical role in the breakdown of αSpII that occurs in FA cells and the reduction in DNA repair. These points and their significance will be discussed below.

### Excessive μ-calpain activity in FA cells leads to increased cleavage of αSpII and decreased DNA ICL repair

**FIGURE 5:**
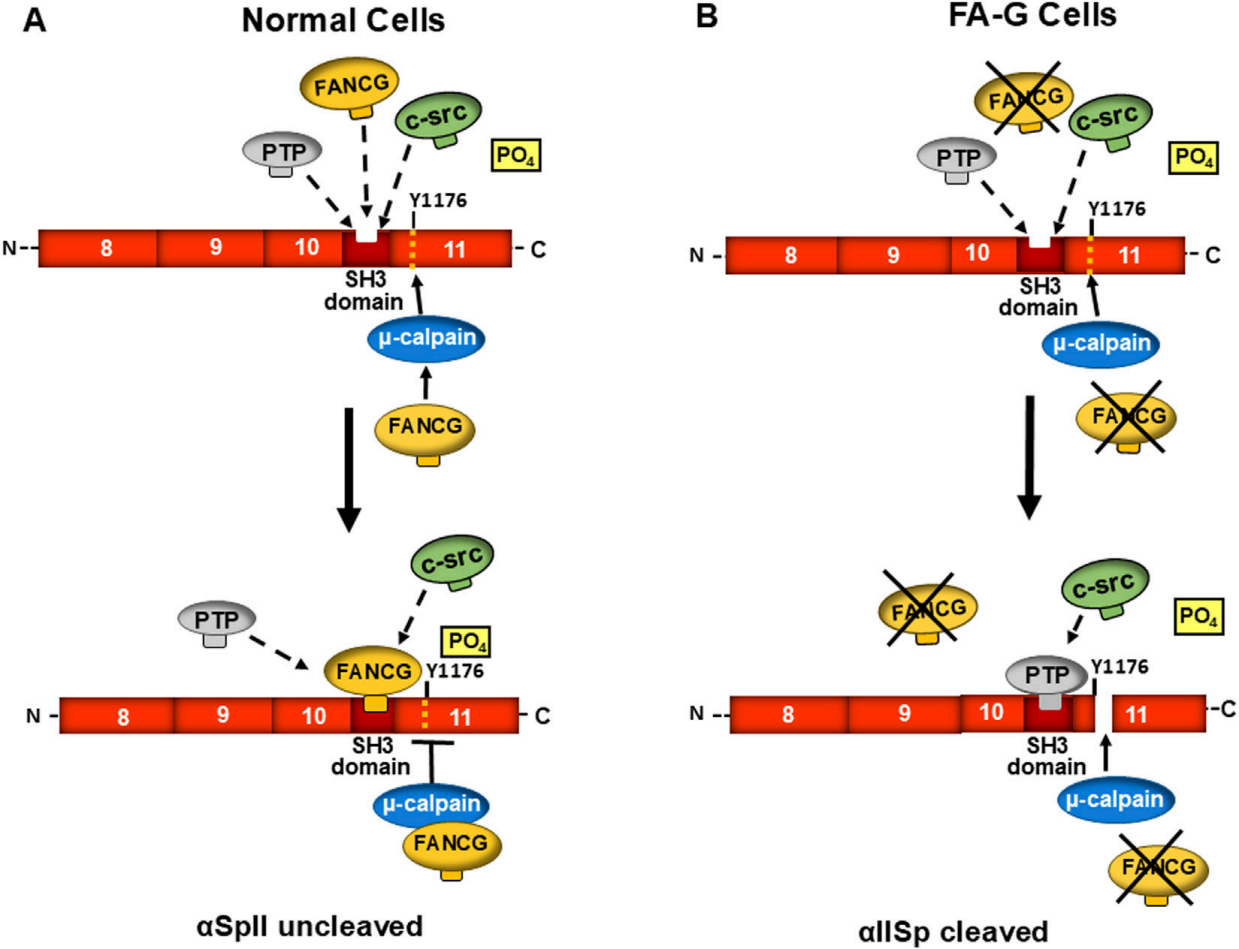
A proposed model for the role of FA proteins in maintenance of stability of αSpII in cells and regulation of its cleavage by µ-calpain. FANCG is used as an example of a FA protein in this process. A segment of αSpII containing repeats 8–11 is represented. **(A)** In normal human cells, an equilibrium exists between low molecular weight phosphotyrosine phosphatase (PTP), FANCG, and c-Src for binding to the SH3 domain of αSpII in repeat 10. When c-Src binds to the SH3 domain of αSpII, it phosphorylates Tyr^1176^ (Y1176) at the adjacent µ-calpain cleavage site. This prevents the ability of µ-calpain to cleave αSpII at its cleavage site. When FANCG binds to the SH3 domain, it prevents PTP binding at this site and μ-calpain cleavage of αSpII. FANCG could also bind to µ-calpain and inhibit its ability to cleave αSpII at this site. In both instances, the inability of µ-calpain to cleave αSpII would enhance the stability of αSpII in the cell. **(B)** In FA cells (FA-G cells for example), a functional FANCG protein is absent and not available for binding to the SH3 domain of αIISp or to µ-calpain. There would be a greater probability that PTP would bind to the SH3 domain. This would lead to the dephosphorylation of Y1176 and allow µ-calpain to cleave αSpII at its cleavage site. FANCG would also not be present to bind to µ-calpain and inhibit its activity. Thus, as is found in FA-G cells, there would be greater cleavage of αIISp. Similar events could occur in different FA complementation groups (e.g., FA-A). (Modified from Zhang et al., [69] with permission from the American Chemical Society).

Within the cell, αSpII is susceptible to cleavage by the protease, μ-calpain [75, 132–134]. αSpII is cleaved by μ-calpain at a single site, Y_1176_-G_1177_, which is adjacent to the SH3 domain of αSpII (Figure 5) [75, 76]. Proteolysis of αSpII by μ-calpain leads to destabilization of the spectrin scaffold and the physiological processes and pathways it regulates [24, 27, 33, 75, 76, 89]. Susceptibility of αSpII to proteolytic cleavage is controlled by phosphorylation/dephosphorylation of residue Y1176 in μ-calpain’s cleavage site [75, 76]. When residue Y1176 is phosphorylated by c-Src, a kinase that binds to the SH3 domain of αSpII, μ-calpain cannot cleave αSpII at its cleavage site [75, 76]. Binding of low-molecular weight phosphotyrosine phosphatase (LMW-PTP) to the SH3 domain of αSpII leads to dephosphorylation of Y1176 and allows μ-calpain to cleave αSpII at this site (Figure 5) [33, 75]. Thus, inhibiting the binding of LMW-PTP to the SH3 domain of αSpII and the subsequent dephosphorylation of Y1176 is important in preventing cleavage of αSpII by μ-calpain. In normal physiological processes, a balance is reached between activation of μ-calpain and substrate-level regulation of αSpII cleavage [75, 76, 135]. This balance depends on maintaining an equilibrium between binding of c-Src and LMW- PTP to the SH3 domain of αSpII, phosphorylation/dephosphorylation of the Y1176 residue and the accessibility of the μ-calpain cleavage site to cleavage by μ-calpain [75, 76].

**FIGURE 6:**
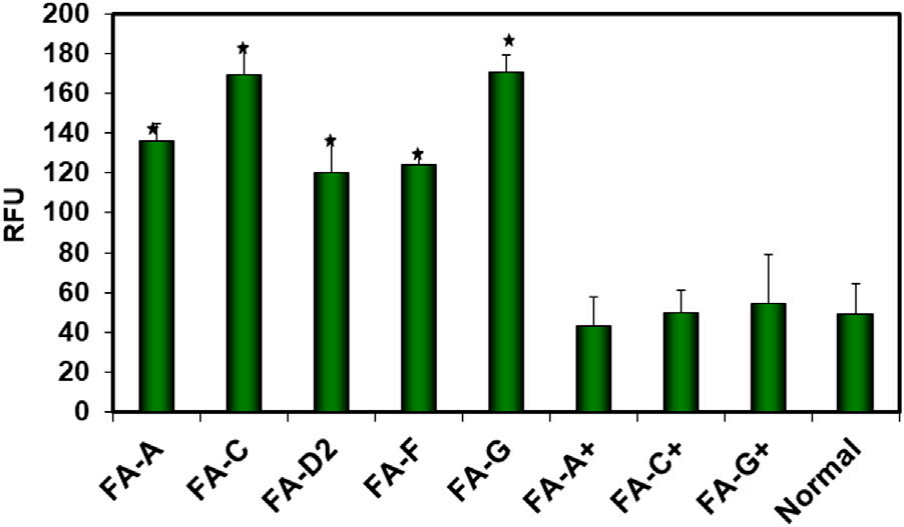
µ-Calpain activity in FA cells. µ-Calpain activity was measured in FA-A, FA-C, FA-D2, FA-F and FA-G cells, in corrected FA-A (FA-A+), FA-C (FA-C+), and FA-G (FA-G+) cells which had been stably transfected with a retroviral vector expressing the appropriate FANC cDNA, and in normal cells. Comparison of µ-calpain activity between the groups of cells showed that µ-calpain activity was 3-4 fold greater in FA cells compared to normal cells or corrected FA cells. Activity was determined by measuring cleavage of a fluorogenic calpain substrate and expressed as relative fluorescence units (RFU). Vertical lines represent ± the standard error of the mean for 5–8 separate experiments (*) (p < 0.0001). (Reproduced from Zhang et al., [69] with permission from the American Chemical Society).

In FA cells, Lambert et al have shown that in a number of complementation groups (FA-A, FA-C, FA-D2, FA-F, and FA-G) μ-calpain activity is 3-4 fold greater than it is in normal cells (Figure 6) [69]. This is not due to an increase in protein levels of μ-calpain, which are similar to those in normal cells, but to the significantly increased activity of μ-calpain [69]. The excessive activation of μ-calpain activity in FA cells is demonstrated by the presence of the characteristic 150 kDa break-down product of αSpII which is produced by μ-calpain proteolytic cleavage of αSpII [69, 132, 140, 141]. This break-down product is relatively stable and widely used as a measure of μ-calpain cleavage of αSpII [141]. The increase in μ-calpain activity in FA cells correlates with decreased levels of αSpII, which are approximately 35–40% of those found in normal cells [13, 38, 44]. That FA proteins play an important role in modulating the levels of µ-calpain activity found in normal cells is demonstrated by the finding that restoring levels of FA proteins in FA cells to those found in normal cells by transfection of FA cells with a retroviral vector expressing the appropriate FANC cDNA leads to reduction of µ-calpain activity to normal levels (Figure 6) [69].

**FIGURE 7:**
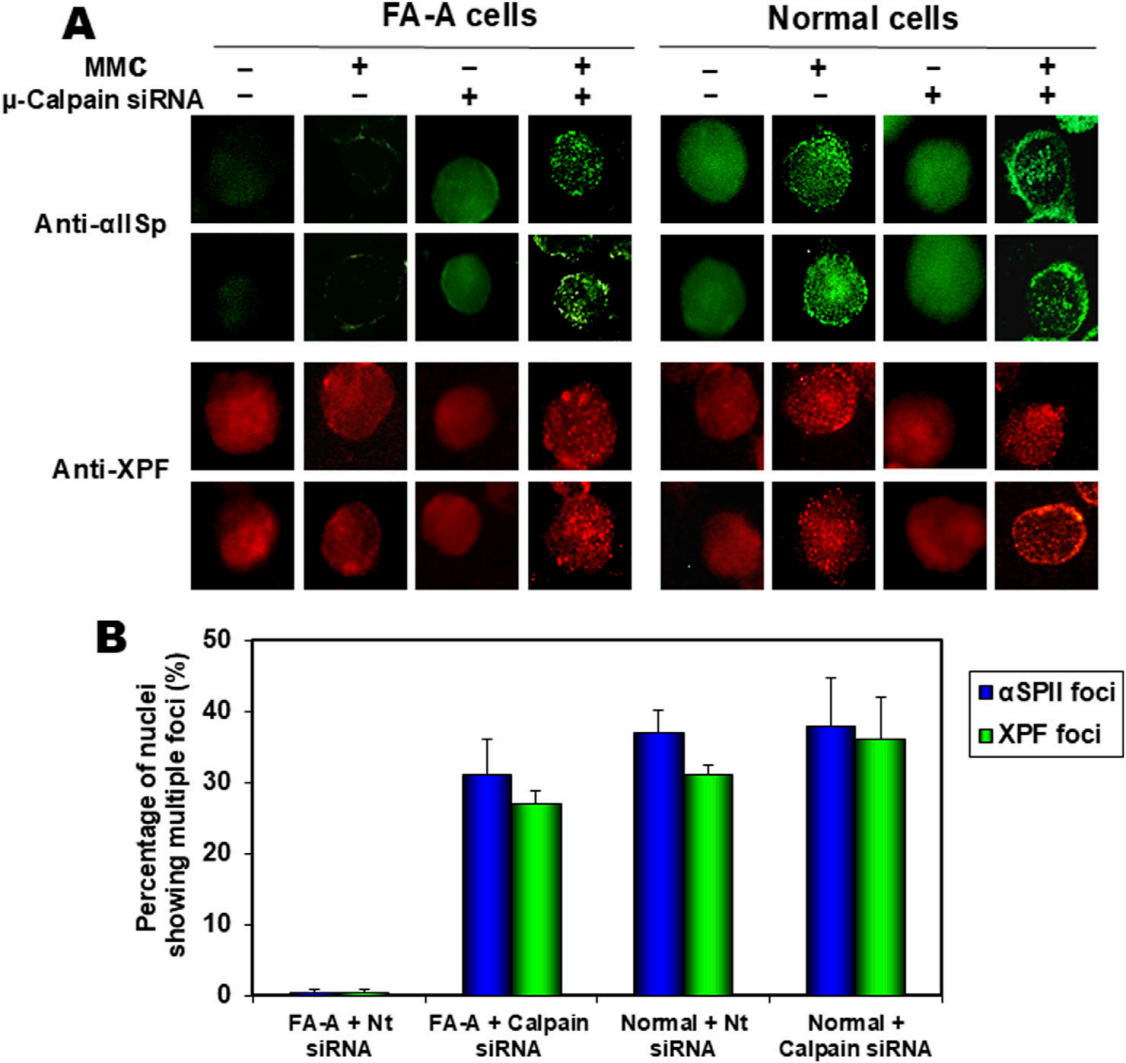
Effect of siRNA knock down of µ-calpain in FA cells on formation of αSpII and XPF nuclear foci after damage with an ICL agent, MMC. **(A)** FA-A and normal cells were transfected with non-target siRNA (−) or µ-calpain siRNA (+) and 24 h after transfection, cells were either undamaged or treated with MMC. αSpII and XPF nuclear foci were examined 16 h after MMC treatment using indirect immunofluorescence and staining with anti-αSpII or anti-XPF. After µ-calpain knock down, αSpII levels in FA-A cells were returned to normal levels and αSpII and XPF localized to damage-induced nuclear foci. **(B)** There was no significant difference in the percentage of nuclei showing multiple foci in FA-A cells in which µ-calpain had been knocked down compared to normal cells transfected with either Nt siRNA or µ-calpain siRNA (Reproduced from Zhang et al., [69] with permission from the American Chemical Society).

Evidence that αSpII cleavage in FA cells is due to increased μ-calpain activity is demonstrated by studies which show that in FA-A cells after knockdown of μ-calpain by siRNA and damage with MMC or 8-MOP plus UVA light, αSpII levels are restored to normal and damage-induced αSpII nuclear foci are observed (Figure 7) [13, 38, 44, 69]. XPF co-localizes with SpII in these foci just as it does in normal cells [69]. This is indicative of DNA ICL repair. In addition, there is a decrease in the chromosomal abnormalities (chromatid beaks, inter chromatid exchanges, chromatid fusions and radial formations) observed in FA cells after DNA ICL damage [69]. Thus, there is strong evidence that increased cleavage of αSpII in FA cells is due to excessive activation of μ-calpain in these cells.

Of particular interest, excessive activation of calpain activity also occurs in a number of neurodegenerative diseases and has been proposed to contribute to the neuropathology of many of these disorders [135]. This excessive activation correlates with a loss of SpII and has been proposed to be associated with the clinical manifestations of these disorders [132, 135, 140, 141]. Excessive activation of calpain and is also found in cancer development [132, 135, 140, 141]. FA may be another example of a disorder in which αSpII breakdown by excessive calpain activity plays an important role in the pathological manifestations of the disorder. This will be discussed in a later section.

### FA proteins play a critical role in reducing cleavage and maintaining the stability of αSpII in cells

FA proteins are proposed to play a critical role in maintenance of αSpII stability and regulation of its cleavage in the cell [13, 50, 69]. In support of this, in corrected FA cells (FA-A, FA-C, and FA-G) which have been stably transduced with a retroviral vector expressing the appropriate FANC cDNA (FANCA, FANCC, or FANCG), FA protein levels return to those found in normal cells, as do the levels of αSpII [13, 50, 69]. In these corrected FA cells, μ-calpain activity is also at normal levels (Figure 6) [69]. Lambert et al have proposed several mechanisms by which FA proteins could aid in maintenance of αSpII stability in the cell and regulation of its cleavage by μ-calpain [13, 50, 69]. One of these is that a FA protein binds to the SH3 domain of αSpII and inhibits the ability of LMW-PTP to bind to the site and dephosphorylate it at Y1176. This, in turn, would prevent the ability of μcalpain to cleave αSpII at its cleavage site and would enhance the stability of αSpII (Figure 5A). In support of this, FANCG has been shown to have a 1@ consensus sequence that directly binds to the SH3 domain of αSpII [101]. This binding could be constitutive. FANCG could exist in equilibrium with c-Src and LMW-PTP for binding to this site. Since affinity of ligands that bind to SH3 domains, in particular the SH3 domain of αSpII, is quite low, the on and off binding rates can be fast, allowing a rapid exchange in binding of these proteins [79, 81]. Importantly, there are a number of patient derived mutations in the *FANCG* gene that result in FANCG having a defect or loss in the consensus sequence that binds to the SH3 domain of αSpII [101]. This could lead to loss of ability of FANCG to bind to αSpII [101]. This would provide LMW-PTP greater accessibility to the SH3 domain and lead to greater cleavage of αSpII by μ-calpain (Figure 5B). FA-G patients have a poorer hematological outcome than patients from other FA complementation groups due to a more severe cytopenia and a higher incidence of AML and leukemia [67, 142]. It would be of interest to determine if this is related to the inability of a defective FANCG to bind to αSpII and aid in preventing excessive cleavage of αSpII by μ-calpain, and in maintaining the stability of αSpII in FA-G cells.

Another possible mechanism by which FA proteins could regulate μ-calpain activity and cleavage of αSpII is that a FA protein binds to μ-calpain and prevents its ability to cleave αSpII. Supporting this is yeast two-hybrid analysis which has shown that FANCA and FANCG bind directly to μ-calpain [69]. This binding could potentially inhibit μcalpain’s activity. Alternatively, a FA protein could bind to αSpII, protecting the μ-calpain cleavage site from attack by μ-calpain. A fourth potential mechanism is that a FA protein could have an effect on the binding of calmodulin to αSpII. Calmodulin binds to αSpII at a site adjacent to μ-calpain’s cleavage site and enhances the susceptibility of αSpII to cleavage by μ-calpain [82–84]. A FA protein, in some manner, could interfere with this binding of calmodulin to αSpII and inhibit its ability to stimulate μ-calpain’s cleavage of αSpII. In FA cells, a defect in a specific FA protein could lead to enhanced binding of calmodulin to αSpII and enhanced cleavage by μ-calpain. FA proteins also bind to each other, particularly those in the FA core group (FANCA, FANCB, FANCC, FANCE, FANCF, FANCG, and FANCL) [143]. For example, FANCG binds directly to FANCF [143]. It is possible that such interactions could aid in association of FA proteins with αSpII.

There are thus a number of potential mechanisms by which FA proteins could play an important role in regulating the activity of μ-calpain and its ability to cleave αSpII. Different FA proteins could affect a different aspect of the αSpII cleavage process, either directly or indirectly. The stability of αSpII is a critical component in the DNA ICL repair process and excessive cleavage of αSpII in FA cells by μ-calpain is proposed to be an important factor in the DNA repair deficiency in these cells [69]. Lambert et al. have proposed that FA proteins play a significant role in the regulation of αSpII cleavage and its stability in the cell [50, 69]. The exact mechanisms by which this occurs needs to be investigated further.

These studies demonstrate another important role for FA proteins in the cell and in the DNA repair process. In addition to the role FA proteins play in DNA ICL repair, it is proposed that they also act as regulators of the cleavage of αSpII by μ-calpain. This could be important for a large number of cellular processes in which αSpII is required, loss of which could lead to chromosome instability, DNA repair defects, congenital and developmental abnormalities, progression of cancer, neuronal degeneration and neurological disorders, which are characteristics of FA.

## αSpII is critical for maintenance of telomere function and chromosome stability after DNA ICL damage

### αSpII recruits repair proteins to telomeres after DNA ICL damage

**FIGURE 8:**
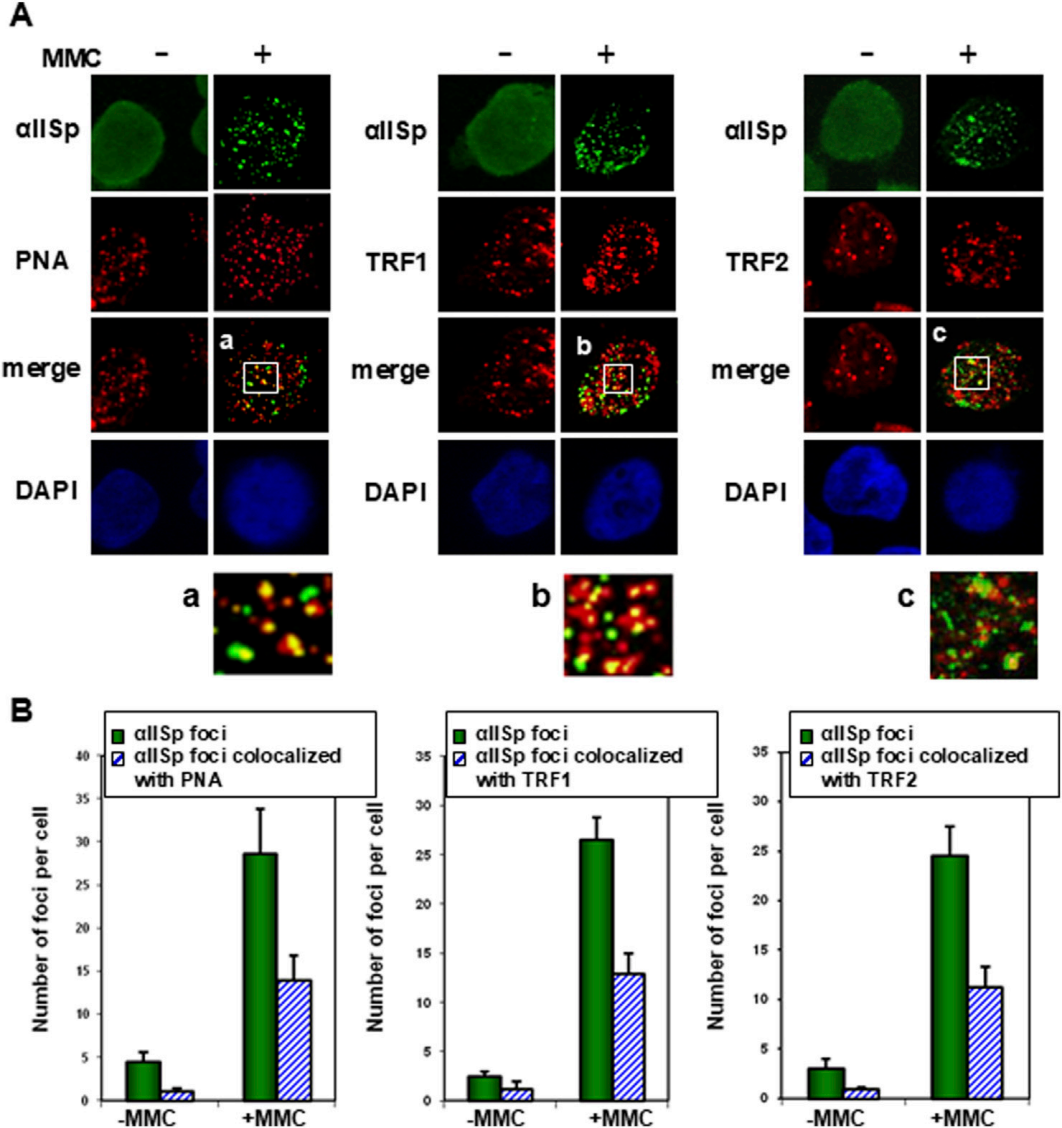
αSpII associates with telomeres after DNA ICL damage. **(A)** αSpII associates with a Cy3-labeled telomere specific probe (PNA) as well as two telomeric proteins in the shelterin complex, TRF1 and TRF2, after 16 h treatment with MMC. Cells were examined using a PNS probe as well as immunoFISH and staining with antispectrin, anti-TRF1 or anti-TRF2 antibodies. Nuclear DNA was counterstained with DAPI. Pictures were taken with Z-stack and one slice is displayed. Magnified images are shown of co-localization of αSpII with (a) PNA probe; (b) TRF1 and (c) TRF2. **(B)** The number of αSpII nuclear foci per cell and the number of αSpII foci that co-localized with PNA, TRF1 and TRF2 foci per cell in normal cells before and after MMC treatment was quantitated. Three hundred cells were counted in each group. Error bars represent SEM. (Modified from Zhang et al., [46] with permission of Oxford University Press).

**FIGURE 9:**
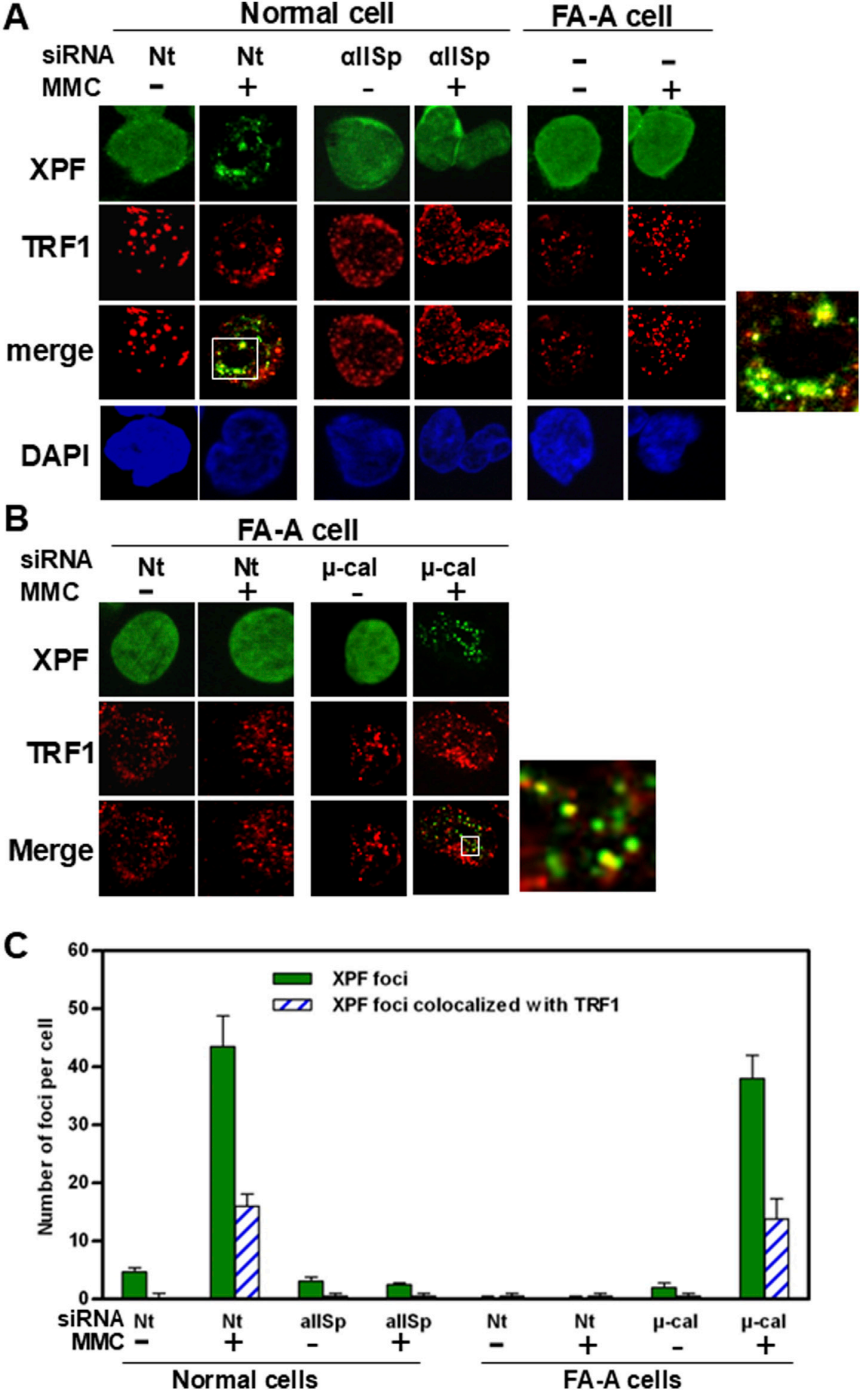
αSpII is needed for recruitment of XPF to sites of ICL damage on telomeric DNA. **(A)** In normal cells transfected with Nt siRNA and treated with MMC for 16 h, XPF co-localized with TRF1. A magnified image of co-localization of XPF with TRF1 in MMC-treated Nt-siRNA-transfected cells is shown to the right. Knock-down of αSpII in normal cells leads to loss of localization of XPF with TRF1 at telomeres after cells are treated with MMC. These studies were carried out using immunoFISH and staining with antiXPF and anti-TRF1 antibodies. Nuclear DNA is counterstained with DAPI. **(B)** In FA-A cells, XPF does not form foci in the nucleus after ICL damage. However, in FA-A cells in which μ-calpain (μ-cal) had been knocked down with μ-calpain siRNA and cells were subsequently treated with MMC, XPF co-localized with TRF1 at telomeres. A magnified image of co-localization of XPF with TRF1 in FA-A cells in which μ-calpain had been knocked down and then treated with MMC is shown on the right. **(C)** The number of XPF nuclear foci and XPF nuclear foci which colocalized with TRF1 was quantitated in normal and FA-A cells. These numbers were the same between normal cells transfected with Nt-siRNA and FA-A cells transfected with μ-calpain siRNA, both of which had been treated with MMC. (Reproduced from Zhang et al., [46] with permission of Oxford University Press).

Telomeres are critical for maintenance of chromosome stability and after DNA damage αSpII plays a major role in this process [50, 144–150]. Lambert et al have demonstrated that αSpII is recruited to telomeres in S phase after DNA ICL damage and is critical for the repair of telomeric DNA at the time when telomeres are undergoing DNA replication [46]. αSpII co-localizes with a telomere-specific Cy [3]-labeled nucleic acid (PNA) oligonucleotide probe and with two proteins in the shelterin complex, TRF1 and TRF2, which help protect the telomere and prevent telomere dysfunction (Figure 8) [46, 50]. These studies were carried out in normal human lymphoblastoid cells which have telomerase, a ribonucleoprotein complex important in maintaining the ends of chromosomes [148, 151–153]. Telomerase positive cells were used since telomerase is present in a number of the cell types involved in the clinical manifestations of FA (e.g., highly proliferating cells, bone marrow cells, peripheral blood cells, stem cells which include hematopoietic stem cells, and numerous types of cancer cells) [154, 155]. These studies have demonstrated that αSpII acts as a scaffold and recruits XPF to sites of ICL damage on telomeric DNA in normal human cells, indicating that it aids in repair of the ICL damage in telomeres [46]. Knock down of αSpII in normal cells leads to loss of localization of XPF to sites of damage on telomeric DNA, demonstrating that αSpII is critical in this recruitment process (Figure 9A) [46]. In FA-A cells, in which there is a deficiency in αSpII, XPF, though present, does not form foci at sites of ICLs on telomeric DNA (Figure 9A). After αSpII levels are restored to normal by knocking down µ-calpain, XPF localizes to sites of damage on telomeres (Figure 9B) [46]. This emphasizes the importance of αSpII in localization of XPF to damage sites on telomeres.

There are differences, however, in the repair events in telomeric DNA compared to non-telomeric DNA. Studies have shown that, in normal human lymphoblastoid cells and HeLa cells, both of which express telomerase, FANCD2, unlike αSpII, does not localize in foci in telomeric DNA after ICL damage [46]. FANCD2 was found, however, to localize to telomeres in immortalized telomerase-negative cells after DNA ICL damage [156]. These studies thus indicate that in telomerase-positive cells FANCD2 is not involved in repair of DNA ICLs at telomeres, however, it is involved in ICL repair in telomeres in telomerase-negative cells. It is possible that, in telomerase-positive cells, the repair response to DNA ICLs is similar to, but not identical to, that which occurs in genomic, non-telomeric, DNA. This needs to be explored further.

### Dramatic reduction of αSpII levels in cells leads to catastrophic loss of telomeres and chromosomal aberrations after DNA ICL damage

**FIGURE 10:**
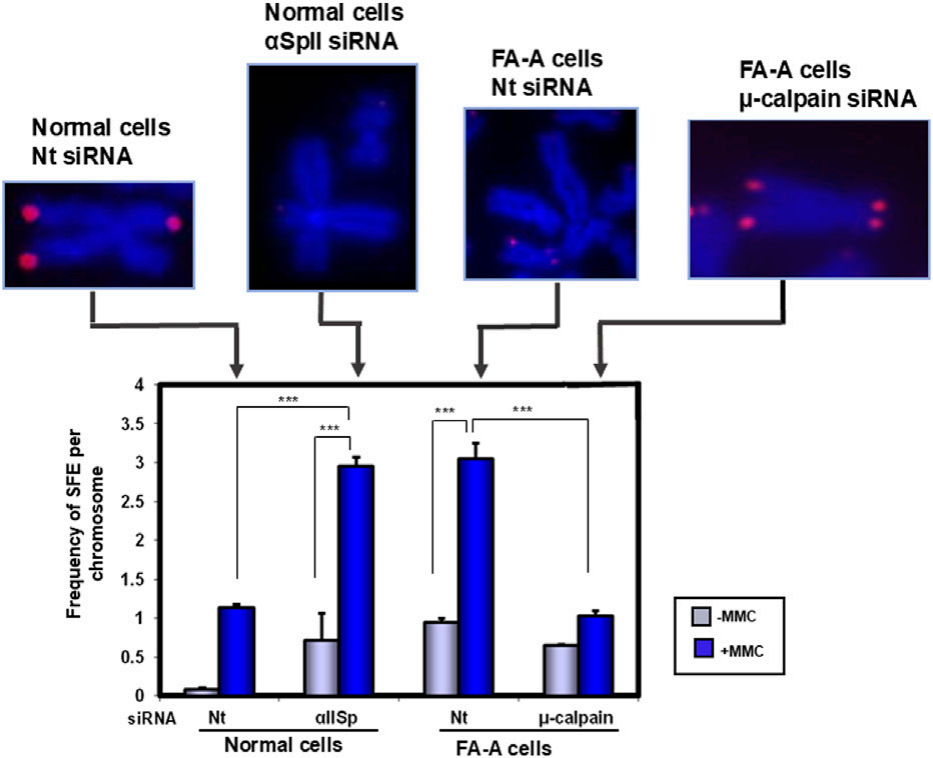
A deficiency in αSpII in cells leads to a catastrophic loss of telomeres after DNA ICL damage. In normal human cells in which αSpII has been knocked down by siRNA, there is a loss of telomeres after treatment with a DNA ICL agent, MMC, compared to normal cells transfected with a Nt siRNA [46]. This is noted by the presence of signal free ends (SFEs) on the chromosomes. In FA-A cells, after damage with MMC, there is a catastrophic loss of telomeres, as noted by the presence of (SFEs), compared to the presence of telomeres on undamaged Nt siRNA transfected FA-A cells [46]. However, in FA-A cells after µ-calpain has been knocked down by siRNA so as to increase αSpII levels, and subsequent treatment with MMC, quantitation of the frequency of SFE in chromosomes showed that it was reduced to levels similar to those in normal cells transfected with Nt siRNA. These studies demonstrate that αSpII has a critical role in maintaining telomere function after damage with a DNA ICL agent [46]. Metaphase chromosomes were stained with DAPI (blue) [46]. Telomeric DNA was identified using FISH with a Cy3-labeled telomere specific PNA probe (red) [46]. Error bars are S.E.M. ***P < 0.0001 (Modified from Zhang et al. [46] with permission from Oxford University Press).

The importance of αSpII in repair of DNA ICLs in telomeric DNA is supported by studies which have demonstrated that, in normal human cells in which αSpII has been knocked-down to levels that are 35% of normal and which have been damaged with a DNA ICL agent, there is telomere dysfunction, as evidenced by the increased formation of telomere dysfunction-induced foci (TIF) [46]. These foci represent sites of DNA doublestrand breaks (DSBs), which arise when the DNA replication forks are stalled at the site of a DNA ICL, and are an indicator of dysfunctional telomeres [46, 157, 158]. It has been proposed that decreased levels of αSpII can lead to stalling of replication of telomeric DNA at sites of DNA ICLs in S phase [46]. This results in collapse of the replication fork and in the formation of DNA DSBs which are measured by determining the accumulation at telomeres of ɣH2AX foci, which are markers for DSBs. In addition to increased TIF formation, knock-down of αSpII also leads to catastrophic loss of telomeres after DNA ICL damage (Figure 10) [46]. This loss can be determined by examination of chromosomes for loss of telomeres or production of signal free ends (SFEs) (Figure10) [46]. Since DNA replication in telomeres occurs in S phase, and αSpII localizes to telomeres in S phase after ICL damage, this suggests that αSpII is important in telomere function during replication of ICL-damaged DNA. A loss of αSpII could thus have significant effects on DNA replication after ICL damage and could lead to production of stalled replication forks. If αSpII is not recruited to these stalled replication forks to aid in repair of the DNA damage, it can be hypothesized that this could lead to failure to efficiently restart replication and to telomere dysfunction and loss of telomeres. These studies demonstrate the importance of αSpII in telomere maintenance and function after DNA ICL damage.

Since chromosome stability depends on functioning telomeres, it is critical that after DNA damage the damage is repaired so that chromosome integrity can be preserved. In normal cells in which αSpII has been knocked down, there is a significant increase in chromosomal aberrations, after DNA ICL damage, which accompanies the decrease in DNA repair that occurs in both non-telomeric and telomeric DNA [45, 46, 69]. These aberrations include: chromatid breaks, sister chromatid end-to-end fusions, radials, and chromosome exchanges [45, 46, 69]. This thus demonstrates that, after DNA ICL damage, αSpII in both non-telomeric and telomeric DNA is critical for maintenance of not only telomere function but also chromosomal stability.

## Telomere dysfunction in FA cells after DNA ICL

FA serves as an excellent model for the effects that a deficiency in αSpII has on telomere function after DNA damage. As mentioned above, levels of αSpII in FA-A cells are approximately 35–40% of normal [12, 45, 48]. Telomere dysfunction has been observed in FA complementation group A (FA-A) cells after DNA ICL (i.e., MMC or psoralen plus UVA light) damage [46]. This includes a significant increase in TIF formation and a catastrophic loss of telomeres (Figure 10) [46]. This increase correlates with loss of αSpII in these cells, which results in a significant decrease in recruitment of XPF to sites of damage at telomeres as mentioned above (Figure 9A) [46]. These studies indicate that there is a defect in repair of ICLs at telomeres in FA-A cells similar to that seen in normal cells after αSpII has been knocked down.

## Knock-down of μ-calpain in FA-A cells corrects the αSpII deficiency, telomere dysfunction, defective DNA repair and chromosome instability after DNA ICL damage

Evidence that loss of αSpII in FA-A cells is a significant factor in the increase in telomere dysfunction and in the catastrophic loss of telomeres that occurs after DNA ICL damage is demonstrated by studies in which μ-calpain has been knocked down by siRNA. Knock down of μ-calpain in FA-A cells leads to restoration of αSpII levels to those found in normal cells and to reduction in the number of TIF positive cells after MMC treatment [46]. In addition, it also results in a significant decrease in the frequency of signal-free ends (SFEs) or telomere loss in FA-A cells after DNA ICL damage (Figure 10) [46]. These studies thus demonstrate that breakdown of αSpII in FA-A cells due to excessive μ-calpain activity results in telomere dysfunction and loss of telomeres, which can be corrected by knocking-down μ-calpain.

In FA-A cells, unlike in normal cells, XPF does not localize to telomeres after DNA ICL damage (Figure 9B) [46]. However, when μ-calpain is knocked down by siRNA and αSpII levels have been restored to normal, XPF co-localizes with αSpII in damage induced foci at telomeres (Figure 9B) [46]. These studies again demonstrate that αSpII plays a critical role in telomere function after DNA ICL damage, and that restoration of αSpII to normal levels in FA cells by knocking down μ-calpain can have a significant effect on increasing the DNA repair capability of these cells and correcting the telomere dysfunction observed after DNA ICL damage [46].

Chromosomal aberrations produced after DNA ICL damage are also corrected in FA cells after restoration of αSpII levels to normal by knocking-down μ-calpain [45, 46]. The aberrations which were corrected include sister chromatid end-to-end fusions, chromosome exchanges, and chromatid breaks and radials and are similar to those produced after ICL damage in normal cells in which αSpII levels have been knocked down [45, 46]. Thus decreasing μ-calpain activity in FA cells and restoring αSpII levels to normal leads to restoration of DNA repair capabilities, localization of XPF to sites of DNA damage, increased chromosomal stability, and a decrease in formation of dysfunctional telomeres and in loss of telomeres after DNA ICL damage. These studies indicate that both loss of αSpII and telomere dysfunction in FA-A cells may be important factors in the chromosomal aberrations which develop after DNA ICL damage.

Of particular interest, FA-A cells in which αSpII levels have been restored to normal are still deficient in the FANCA protein. The question that arises is how DNA ICL repair can still proceed in the absence of this protein. Lambert et. al. have proposed that FA proteins, in addition to a role in DNA repair, are also important in maintaining the stability of αSpII and reducing its cleavage by μ-calpain [46, 69]. When αSpII stability has been restored in FA-A cells by knocking-down μ-calpain, DNA repair is able to proceed, possibly involving an alternative means [49, 50]. In FA-A cells, in which there is a deficiency in FANCA, FANCD2 is not monoubiquitinated [49]. FANCD2-Ub has been shown to be important for the ICL repair pathway to proceed [57–59]. Lambert et al have shown, however, that in FA-A cells in which αSpII levels have been restored, non-Ub FANCD2 foci are observed at levels approximately 80% of normal just as they are in FA-A cells in which levels of αSpII have not been restored [49]. FANCD2-Ub is not present [49]. The non-Ub FANCD2 foci localize to sites of DNA ICL damage and the nonUb FANCD2 foci follow the same time course for formation of nuclear foci after ICL damage as the FANCD2-Ub foci do. However, in the FA-A cells non-Ub FANCD2 foci only appear after levels of αSpII have been returned to normal [49]. Thus non-Ub FANCD2 requires the presence of αSpII in order to localize to sites of damage. αSpII does not colocalize with these non-Ub FANCD2 foci, just as it does not co-localize with FANCD2- Ub foci in normal cells after DNA ICL damage [49]. Lambert et al have proposed that in FA-A cells when levels of αSpII have been returned to normal, non-Ub FANCD2 localizes to sites of damage and plays a role in the DNA repair process [49]. This is potentially an alternate method for DNA ICL repair. Whether this is mainly a back-up mechanism or is also involved in normal ICL repair is not clear. Other studies support the proposal that non-Ub FANCD2 plays a role in DNA ICL repair, in particular, in the nonhomologous recombination step in the repair process and in replication fork recovery [159–163]. The relationship between αSpII and FANCD2-Ub and non-Ub FANCD2 after DNA damage needs to be further investigated.

## Importance of loss of αSpII in the pathogenesis of Fanconi anemia

As noted above, breakdown and loss of αSpII in FA cells has a significant effect on both DNA repair and chromosome stability after damage with a DNA interstrand crosslinking agent. Lambert et al. have proposed that this loss is an important contributing factor in the pathogenesis of FA [38, 44]. There are a number of defining characteristics associated with FA pathogenesis, the major ones of which include a defect in DNA repair, chromosome instability, cancer predisposition, bone marrow failure and multisystemic congenital abnormalities [47, 51–69]. Experimental evidence has shown that loss of αSpII in FA cells has a significant impact on several of these characteristics. Breakdown of αSpII in FA cells has been shown to be directly involved in the defect in ability of FA cells to repair DNA ICLs [13, 44, 45]. Loss of αSpII results in failure of XPF/ERCC1 to localize to sites of DNA ICLs and create incisions at these sites leading to defective DNA ICL repair in FA cells in both telomeric and non-telomeric DNA [13, 44, 47, 50]. In addition, excessive breakdown of αSpII in FA cells also results in chromosome instability and production of chromosomal aberrations after DNA ICL damage [44, 45]. It has been shown that failure to repair DNA ICLs, particularly in S phase of the cell cycle can directly lead to the production of DNA double-strand breaks and chromosomal aberrations [164]. This is proposed to be a major cause of chromosomal aberrations in FA cells after DNA ICL damage [44, 45, 50]. Restoration of αSpII levels to normal in FA cells, by knocking down μ-calpain, leads to restoration of DNA ICL repair and chromosome stability [45, 47, 50]. These studies thus indicate that αSpII is critical for two important cellular processes, DNA repair and chromosome stability of DNA ICL damage, which are defective in FA cells and which contribute to the pathogenesis of the disorder.

Another phenotypic characteristic of FA is an increased incidence of cancer (e.g., acute myeloid leukemia (AML) and squamous cell carcinoma) and hemolytic anemia [67, 68, 77, 165]. There is evidence that a deficiency in αSpII plays an important role in cancer development [68, 166]. Loss of αSpII has been reported in the bone marrow of patients with acute myeloid leukemia (AML) and it has been proposed that a deficiency in αSpII is a contributing factor in the development of AML [165, 167]. Since FA patients can develop AML, it is possible that loss of αSpII is an important component in this process and may be involved in the leukemogenesis and bone marrow failure which can occur [165, 167]. In a number of B-cell malignant lymphomas, there is a strong correlation between loss of αSpII and development of lymphomagenesis [167]. This has led to the suggestion that a deficiency in αSpII is an important factor in lymphomagenesis [167]. Thus breakdown and loss of αSpII in FA cells could play a role in the development of AML and leukemogenesis observed in FA patients and contribute to FA pathogenesis. This needs to be further examined.

FA patients can also have a number of congenital abnormalities, which include radial ray deformities, absent radi, ear malformations, urogenital anomalies, renal deformities, cardiac defects, and neurological abnormalities [67, 168, 169]. αSpII has been proposed to play a role in many developmental processes [36, 170–172]. In αSpII knockout mice the embryos display cardiac, craniofacial and neural tube abnormalities [170]. Cultured fibroblasts from these mice have impaired growth and spreading [170]. These studies indicate that αSpII is important in cellular morphology and development. Since αSpII is critical for and expressed throughout development in mammalian cells, it can be hypothesized that a deficiency in αSpII in FA patients could indirectly have a significant influence on a number of the developmental abnormalities associated with FA.

## Other disorders associated with defects in αSpII

αSpII is an essential protein in the cell and an important cellular scaffold which is critical for a diverse number of biological processes, therefore, loss or dysfunction of this protein could be of important clinical significance and play a fundamental role in the etiology of a number of diseases [7, 13–17, 25–39]. Two important causes for a loss or deficiency in αSpII are: (1) enhanced cleavage and break-down of αSpII, or (2) mutations in the gene encoding αSpII, *SPTAN1,* resulting in expression of a defective αSpII. Some disorders in which defects or a deficiency in αSpII are of clinical significance are presented below.

Loss or a deficiency in αSpII is an important factor in the pathophysiology of a number of neurological disorders [135, 169, 173–179]. Mutations in the *SPTAN1* gene are associated with a broad range of neurodevelopmental diseases [169, 174, 175]. One of these is early infantile epileptic encephalopathy, or West Syndrome, which is characterized by progressive brain atrophy, severe neurodevelopmental impairment, mental retardation, difficulty walking, and seizures [169, 174–177]. In this disorder, there is a mutation in the *SPTAN1* gene leading to a defect in the binding of αSpII to βSpII and to βSpIV, which are needed for organization and maintenance of the neuronal actin-spectrin cytoskeleton, as well as for axonal and dendritic growth and development and neuronal excitabiliity [174–177].

In a number of other progressive neurodegenerative disorders there is increased breakdown of αSpII [132, 135, 140, 141, 178–181]. It has been shown that this is due to excessive activation of calpain which results in increased cleavage of αSpII. Excessive activation of calpain and αSpII cleavage is a common feature of neurodegenerative diseases and of traumatic encephalopathy and leads to loss of neuronal integrity and the neuropathology of many of these disorders [132, 135, 140, 141, 178, 179]. In Alzheimer’s disease, increased breakdown of αSpII has been demonstrated in numerous cells and tissues and is due to excessive calpain activity but not to changes in levels of calpain [141, 171, 177, 182]. It has been proposed that faulty regulation of the neuronal spectrin skeleton and its breakdown by excessive calpain activity can activate a series of events that leads to the ataxia and neuronal degeneration observed in this and other neurodegenerative disorders [135]. Thus, excessive activation of calpain activity in a number of neurodegenerative diseases results in loss of αSpII [132, 135, 140–142].

Central nervous system and various neurological anomalies are also a clinical characteristic of FA patients. These include: brain and spinal cord anomalies, microcephaly, hydrocephalus, cerebellar defects, and development of medulloblastoma [67, 168, 173, 183–186]. Of considerable interest, FA cells have significantly (3-4 fold) increased levels of breakdown of αSpII due to an excessive increase in µ-calpain activity [13, 50, 69]. FA thus represents another disorder in which loss of αSpII is due to its cleavage by µ-calpain. It can be hypothesized that like some neurodegenerative diseases, excessive activation of μ-calpain activity in FA, leading to significant cleavage of αSpII, may be an important contributing factor in the pathological changes observed in the nervous system and in the pathogenesis of FA. It would be of interest to determine whether there is increased μ-calpain activity and αSpII cleavage in neuronal tissue in FA patients with neurological abnormalities and to speculate that, if a relationship between these two factors were found, this would indicate that breakdown of αSpII is an important factor in progression of these abnormalities.

There is evidence that a deficiency in αSpII plays an important role in development of cancers other than AML. In Lynch Syndrome or hereditary nonpolyposis colorectal cancer, where there is a defect in DNA mismatch repair and a deficiency in the mismatch repair protein, MLH1, there is a significant reduction in levels of αSpII in the tumor cells [187–189]. Since MLH1 directly interacts with αSpII, it has been proposed that loss of MLH1 leads to destabilization of αSpII which, in turn, results in a defect in DNA repair and in the tumor development seen in this disorder [187–189]. These studies, along with those noted above, which suggest that αSpII loss is an important factor in lymphomagenesis, collectively indicate that there is strong evidence that a deficiency in αSpII plays an important role in development of a number of different types of cancer.

In both acquired and neonatal congenital heart failure, there is dysfunction of αSpII that is due to excessive calpain activity which results in increased cleavage of αSpII [190, 191]. This has been demonstrated by the presence of the 150 kDa αSpII breakdown product of μ-calpain in the cardiac cells [190, 191]. αSpII is required for normal cardiac structure, function and development and plays a central role in the formation and regulation of key structural and signaling pathways in the heart [190, 191]. Breakdown of αSpII, which can occur in heart disease, has a significant effect on the cardiomyocyte spectrin network and on regulation of normal cardiac function [36, 190, 191]. Thus, breakdown of αSpII by excessive μ-calpain activity is also an important factor in heart failure.

αSpII dysfunction can therefore lead to a variety of disorders, particularly since it is a multifunctional protein which is critical for a multitude of diverse cellular processes. As mentioned above, there is a relationship between clinical manifestations of all of the disorders described and a loss or deficiency in αSpII, which in turn is due to either enhanced cleavage of αSpII by excessive proteolytic cleavage by μ-calpain or expression of a defective αSpII due to a mutation in the *SPTAN1* gene. As a result of the diversity of αSpII function, a deficiency in αSpII can lead either directly or indirectly to dysregulation of a number of different cellular pathways and to pathological changes which are manifested in a variety of different disorders. The number of disorders in which deficiencies in αSpII play a direct or indirect role may still not yet be fully recognized.

## Conclusions and perspective

αSpII is a critically important and functionally diverse protein which represents 2–3% of all proteins in human cells [14–17]. It forms a network under the plasma membrane and within the cytoplasm ensuring the stability of cell membranes and organelles. It acts as a scaffold interacting with different protein binding partners playing an important role in cell development, cell migration, cell cycle, actin filament organization, intracellular trafficking and signal transduction [24–28]. Within the nucleus, αSpII is part of a nucleoskeletal network which is essential for maintaining the structural integrity of the nuclear envelope and elasticity of the nucleus. It is involved in the biomechanical coupling of the nucleoskeleton to the cytoskeleton. Of particular significance, αSpII plays a critical role in DNA repair where it acts as a scaffold recruiting repair proteins to sites of damage, enabling DNA repair to take place [44, 50].

αSpII’s stability within the cell is critical for all of its functions. It is sensitive to cleavage by the protease, μ-calpain, and excessive activation of μ-calpain activity leads to increased cleavage of αSpII and loss of its ability to function in essential cellular processes, which is a contributing factor in a number of disorders [44, 50]. Maintaining a balance between activation of μ-calpain and regulation of spectrin cleavage is thus extremely important. This is particularly critical during the repair of DNA interstrand crosslinks (ICLs), where αSpII plays a critical role in repair of damaged sites, on both telomeric and non-telomeric DNA [46]. Excessive activation of µ-calpain activity leads to cleavage and breakdown of αSpII and loss of its ability to function in DNA repair as well as in cellular systems critical for both the structural and non-structural components of cell function [69]. This, in turn, leads to development of the pathological changes observed in numerous disorders.

**FIGURE 11:**
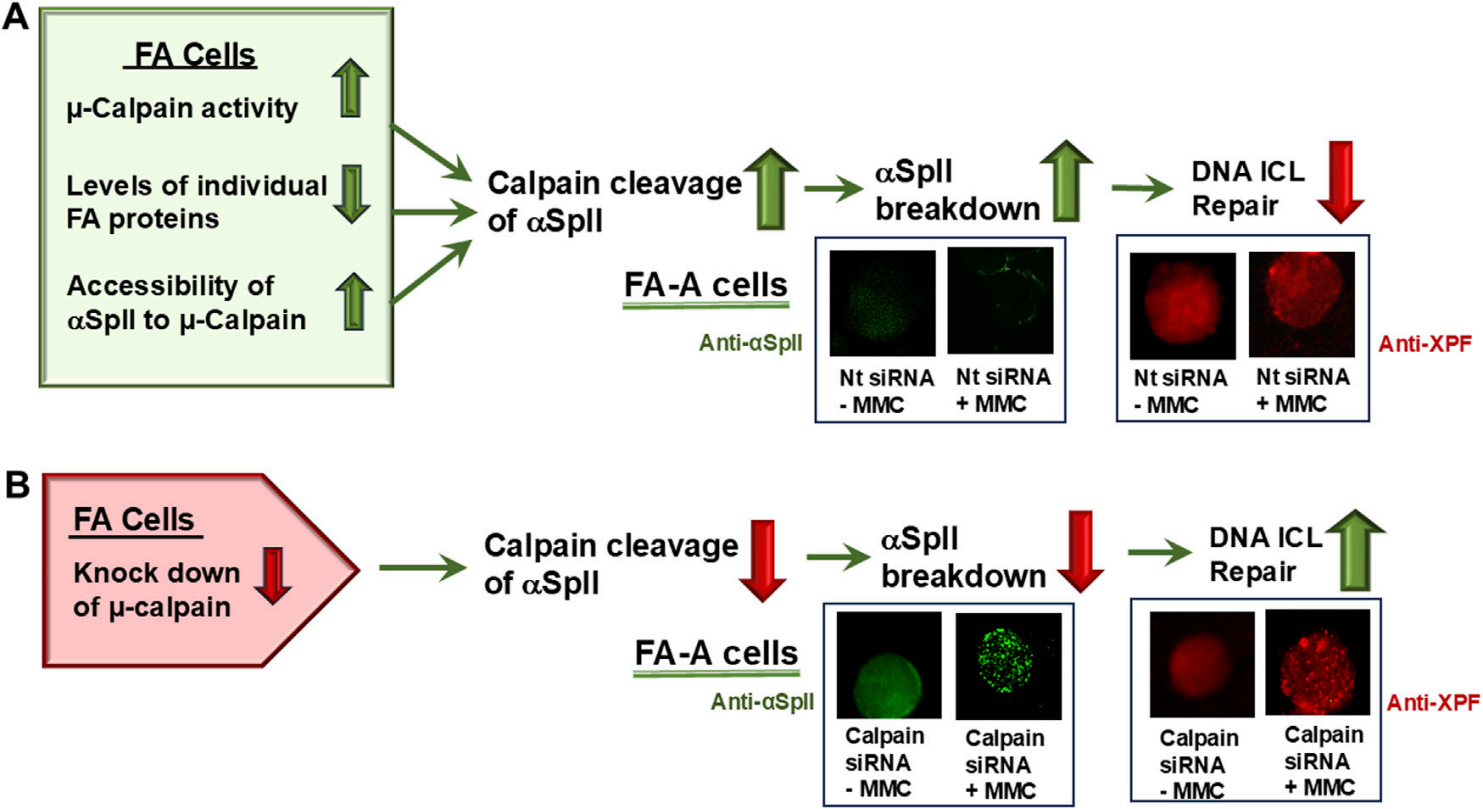
Role of µ-calpain in the deficiency of αSpII in FA cells and in defective DNA ICL repair in these cells. FA-A cells are used as a model. **(A)** In FA-A cells, excessive µ-calpain activity, decreased levels of individual FA proteins and increased accessibility of αSpII to µ-calpain lead to increased cleavage of αSpII by µ-calpain. FA-A cells, transfected with a non-target siRNA and examined using indirect immunofluorescence and staining with anti-αSpII, show low levels of αSpII and few nuclear foci after MMC treatment. Staining of the cells with anti-XPF shows that XPF is present in these cells but, after MMC treatment, few XPF foci are seen. **(B)** In FA-A cells, in which µ-calpain has been knocked down by µ-calpain siRNA, there is decreased cleavage of αSpII. Examination of these cells using immunofluorescence shows that αSpII is present in the nucleus and forms foci after treatment with MMC. XPF also forms nuclear foci in these cells after MMC treatment.

One disorder in which there is a deficiency in αSpII is the bone marrow failure disorder, Fanconi anemia (FA), a distinctive hallmark of which is a defect in ability to repair DNA ICLs. αSpII levels in FA cells are 35%–40% of normal and levels of repair are 35–45% of normal [43, 48, 54]. Of particular significance, loss of αSpII in FA cells is due to increased µ-calpain activity, which is 3-4 fold greater than in normal cells, as observed in FA-A, FA-C, FA-D2, FA-F and FA-G cells [69]. This leads to increased cleavage of αSpII and decreased DNA ICL repair capabilities in both telomeric and non-telomeric DNA (Figure 11A). However, when levels of µ-calpain are knocked down, there is decreased cleavage of αSpII, whose levels return to those found in normal cells, and restoration of DNA ICL repair in these cells (Figure 11B). Another characteristic hallmark of FA is chromosome instability. After DNA ICL damage, a number of chromosomal aberrations occur in FA cells such as sister chromatid end-to-end fusions, chromatin breaks, chromosome exchanges and radials [45]. In FA cells in which µ-calpain has been knocked down, αSpII stability is restored leading to restoration of chromosomal stability and a decrease in chromosome aberrations after DNA ICL damage. This indicates that αSpII plays an important role in both DNA repair and chromosome stability after DNA ICL damage.

Lambert et al. have hypothesized that excessive cleavage of αSpII in FA cells is an important contributing factor in the pathogenesis of this disorder [44, 50]. The strongest evidence supporting this is from the studies noted above on the critical role of αSpII in DNA repair and in chromosome stability, since defects in these two processes are of major importance in FA pathogenesis. αSpII has been shown to play a direct role in DNA ICL repair and in the repair defect in FA. It binds directly to DNA containing ICLS and its involvement in the repair process has been demonstrated both by measurement of unscheduled DNA synthesis (UDS) after damage, which measures uptake of nucleotides into DNA repair patches, and by localization of the repair protein, XPF, to sites of DNA ICLs in normal and FA cells [42, 43, 47]. These studies indicate that breakdown of αSpII in FA cells is directly related to the defect in DNA ICL repair. An important question is what are the factors that are important in maintaining the stability of αSpII in FA cells. Studies have demonstrated that one of these is regulation of cleavage of αSpII by μ-calpain.

Excessive cleavage of αSpII by µ-calpain and its involvement in the DNA repair defect in FA cells has been demonstrated both by the presence of the 150 Kd αSpII breakdown product of µ-calpain in FA cells and by studies which show that knocking down μ-calpain corrects the excessive cleavage of αSpII and the DNA repair defect [69]. This needs to be examined in more FA complementation groups. It will also be important to determine whether there are other proteins involved in αSpII cleavage. Calmodulin, for example, binds to a site on αSpII and can stimulate µ-calpain activity. Studies need to be carried out to determine whether increased binding of calmodulin to αSpII in FA cells plays a role in the increased µ-calpain activity observed and the increased breakdown of αSpII.

FA proteins are known to play a role in the DNA ICL repair pathway. Lambert et al. have hypothesized that they also play an important role, either directly or indirectly, in maintaining αSpII stability. Transfection of FA cells (FA-A, FA-C, and FA-G) with the corresponding FANC-cDNA, which results in expression of either FANCA, FANCC or FANCG, respectively, restores αSpII stability and leads to restoration of chromosomal stability [69]. FANCG has been shown to directly bind to the SH3 domain in αSpII [101]. It has been proposed that FANCG may be involved in aiding in maintenance of αSpII stability by directly preventing binding of low molecular weight phosphotyrosine phosphatase (LMW-PTP) to its SH3 domain, which would lead to dephosphorylation of residue Y1176 at μ-calpain’s cleavage site adjacent to the SH3 domain (Figure 1) and allow µcalpain to cleave αSpII at this site [101]. Binding of FANCG to the SH3 domain would aid in preventing cleavage of αSpII by μ-calpain. This needs to be further investigated. Other FA proteins could also interact with αSpII to protect it from cleavage by µ-calpain or bind to µ-calpain or calmodulin and prevent their activity, either directly or indirectly, via other proteins. FANCA and FANCG, for example, have been shown to bind directly to μcalpain [13, 50]. Whether this binding can decrease the activity of µ-calpain and enhance αSpII stability needs to be examined. Similarly, binding of a FA protein to calmodulin could inhibit its ability to bind to μ-calpain and activate it, which would also enhance αSpII stability. Thus, binding of FA proteins to αSpII or to associated proteins such as μcalpain or calmodulin could have an important effect on αSpII stability, protecting it from cleavage by µ-calpain. Studies need to be carried out on all of these possibilities.

Numerous studies have been done on the direct role FA proteins play in repair of DNA ICLs. In one of these, knockdown of the *FANCA* and *FANCD2* genes in human embryonic stem cells was shown to lead to a marked reduction in localization of FANCA and FANCD2 proteins to ICL damage induced foci, indicating presence of a defect in DNA ICL repair [192]. Chromosomal aberrations such as breaks and radials also formed [192]. It would be interesting to determine, in studies such as these, whether a decrease in FANCA or FANCD2 proteins leads not only to direct effects on DNA ILC repair but also to loss of stability and breakdown of αSpII. If this occurred, it would suggest that loss of the FA protein led to increased breakdown of αSpII and to the increased defect in DNA repair and chromosome stability observed after DNA ICL damage. These studies would also emphasize the importance of FA proteins in aiding in maintenance of the stability of αSpII in cells, as has been suggested.

Another distinctive hallmark of FA is chromosome instability. The importance of αSpII in maintenance of chromosome stability is seen in studies in which knock down of αSpII in normal cells leads to production of chromosomal aberrations [45]. In addition, in FA cells when levels of αSpII are returned to normal by knocking down μ-calpain, chromosome stability is restored indicating that αSpII plays a role in this process [45]. The effect that excessive cleavage of αSpII in FA cells has on chromosome stability could be directly related to the defect in the ICL repair process and the increase in un-repaired DNA which is produced in these cells. It has been shown that un-repaired or misrepaired lesions in DNA can lead to chromosomal aberrations [164]. Cells are particularly sensitive to un-repaired DNA ICLS, which can lead to DNA double-strand break formation in S phase of the cell cycle and to chromosomal aberrations [164]. In FA cells, breakdown of αSpII could thus be important in production of the chromosomal aberrations observed, which particularly occur after DNA ICL damage and which are not effectively repaired in these cells.

Lambert et al. have also hypothesized that excessive cleavage of αSpII in FA cells may be an important contributing factor in some of the other characteristics of FA pathogenesis such as bone marrow failure, development of AML, congenital anomalies and neurological abnormalities [13, 44]. Studies have shown, for example, that there is a loss of αSpII in bone marrow of patients with AML and it has been proposed that this loss may be contributing to AML development and to leukemogenesis [165, 167]. It can be hypothesized that since FA patients can develop AML, a deficiency in αSpII is a contributing factor in its development. Excessive cleavage and loss of αSpII also occurs in a number of neurodegenerative disorders. This is due to excessive activation of calpain and is a common feature of the neuropathology of many of these disorders. Neurological abnormalities are also a characteristic of FA patients [67, 168, 183–186]. It is possible that excessive calpain activity may thus be an important factor contributing to the loss of αSpII and to a number of the neurological anomalies observed in FA. FA could thus represent another disorder in which there is excessive cleavage of αSpII by μ-calpain. Studies needs to be carried out on this in more complementation groups of FA. Since αSpII is a multifunctional protein, it is not surprising that a deficiency in it could lead to deficiencies in multiple cellular pathways and result in a number of the different pathological manifestations observed in FA.

This review has focused on the important role of αSpII in repair of DNA ICLs in telomeric and non-telomeric DNA and on the importance of maintaining the stability of αSpII not only for DNA repair but also for other cellular processes. Two factors which have been shown to be critical for maintaining αSpII stability in the cell are the levels of μ-calpain activity and the presence of FA proteins. Excess μ-calpain activity in FA cells has been demonstrated to lead to breakdown of αSpII. FA proteins are proposed to play a role in regulation of αSpII stability by modulating its cleavage by μ-calpain. Direct binding of FANCG to αSpII has been demonstrated but the effects this binding has on αSpII stability and its cleavage by μ-calpain needs to be further investigated as does the effect of other FA proteins on this process.

Since αSpII has a wide spectrum of functions in a number of different cellular and physiological processes, it is possible that in FA cells excessive cleavage of αSpII by μ-calpain could have an important effect on some of the clinical characteristics of this disorder. Decreasing μ-calpain activity in FA cells could aid in stabilization of αSpII and be of potential therapeutic relevance. Lambert et al. have shown that knocking down μcalpain activity in FA cells to levels found in normal cells does not have any adverse effects on cell survival and it leads to restoration of αSpII to normal levels and enables DNA ICL repair to proceed. Though levels of μ-calpain have been reduced to normal in these cells, the deficiency in the FA protein specific for the FA complementation group being examined has not been corrected. However, DNA repair takes place. FANCD2-Ub is not present but non-Ub FANCD2 is and it localizes in foci at sites of damage. αSpII is needed for this to occur [49]. αSpII localizes in foci at sites of damage along with XPF but not with non-Ub FANCD2. It has been proposed that this could be a backup system in which non-Ub FANCD2 is involved in the repair process [49]. In these FA cells, if a specific FA protein is not available to help stabilize αSpII, restoration of αSpII to normal levels by knocking down μ-calpain could, to some extent, counteract this need for the FA protein in this process. Whether this holds true for all of the FA complementation groups needs to be examined.

It is hypothesized that there is a mechanistic link between excessive μ-calpain activity, cleavage of αSpII, and defective DNA ICL repair, and that these are key factors in the pathogenesis of FA. FA proteins are proposed to play a role in modulating αSpII cleavage by μ-calpain. Correcting the excessive cleavage of αSpII in FA cell is particularly important since αSpII is essential for a number of critical cellular and physiological processes, such as DNA repair. These studies suggest a new direction for correction of a number of the phenotypic deficiencies in FA cells. Thus, stabilization of αSpII in FA by reducing μ-calpain activity, potentially in combination with other modalities, could potentially reduce the DNA repair defect and other systemic deficiencies which lead to the clinical manifestations of this as well as other disorders.
